# Characterization of the Phosphofructokinase Gene Family in Rice and Its Expression Under Oxygen Deficiency Stress

**DOI:** 10.3389/fpls.2013.00125

**Published:** 2013-05-14

**Authors:** Angelika Mustroph, Johanna Stock, Natalia Hess, Sophia Aldous, Anika Dreilich, Bernhard Grimm

**Affiliations:** ^1^Department of Plant Physiology, University of BayreuthBayreuth, Germany; ^2^Department of Plant Physiology, Institute of Biology, Humboldt-University BerlinBerlin, Germany

**Keywords:** *Oryza sativa*, anoxia, submergence, phosphofructokinase, pyrophosphate

## Abstract

Plants possess two types of phosphofructokinase proteins for phosphorylation of fructose-6-phosphate, the ATP-dependent phosphofructokinase (PFK) and the pyrophosphate-(PPi) dependent pyrophosphate-fructose-6-phosphate-phosphotransferase (PFP). During oxygen deficiency ATP levels in rice seedlings are severely reduced, and it is hypothesized that PPi is used as an alternative energy source for the phosphorylation of fructose-6-phosphate during glycolysis. In this study, we analyzed the expression of 15 phosphofructokinase-encoding genes in roots and aerial tissues of anoxia-tolerant rice seedlings in response to anoxic stress and compared our data with transcript profiles obtained from microarray analyses. Furthermore, the intracellular localization of rice PFK proteins was determined, and the PFK and PFP isoforms were grouped in a phylogenetic tree. Two *PFK* and two *PFP* transcripts accumulated during anoxic stress, whereas mRNA levels of four *PFK* and three *PFP* genes were decreased. The total specific activity of both PFK and PFP changed only slightly during a 24-h anoxia treatment. It is assumed that expression of different isoforms and their catalytic properties differ during normoxic and anoxic conditions and contribute to balanced glycolytic activity during the low-oxygen stress. These characterizations of *phosphofructokinase* genes and the comparison to other plant species allowed us to suggest candidate rice genes for adaptation to anoxic stress.

## Introduction

The pronounced tolerance of oxygen deprivation during seed germination and vegetative development makes rice plants an interesting subject to elucidate mechanisms of response to low-oxygen stress. Oxygen deficiency results in inhibition of mitochondrial respiration, leading to NADH accumulation as well as ATP deficiency. Glycolytic production of ATP continues, when fermentative enzymes are induced and able to regenerate sufficient amounts of NAD^+^ (reviewed in Drew, 1997; de Sousa and Sodek, 2002; Geigenberger, 2003; Gibbs and Greenway, 2003).

Plant cells frequently enhance the rate of sucrose consumption under low-oxygen stress to compensate for the low energy yield during glycolysis (2–4 mol ATP per mol glucose), in comparison to aerobic mitochondrial respiration (30–36 mol ATP per mol glucose) (Summers et al., 2000). We have shown previously that rice plants sustain a higher fermentation rate during anoxia as compared to anoxia-sensitive wheat plants, but ATP levels still drop by two-third (Mustroph et al., 2006a). The decline in cellular ATP content during low-oxygen stress will impact the glycolytic phosphorylation reactions catalyzed by hexokinases and phosphofructokinases (Bouny and Saglio, 1996). Thus, it was hypothesized that plants might use pyrophosphate (PPi) instead of ATP as an alternative energy source for phosphorylation processes during ATP deficiency (Weiner et al., 1987; Stitt, 1998; Huang et al., 2008). PPi is a by-product of many biosynthetic processes like DNA and protein synthesis, and its levels are not changed during oxygen deficiency stress (Dancer and ap Rees, 1989; Mohanty et al., 1993; Mustroph et al., 2005).

Sucrose cleavage and subsequent phosphorylation of hexoses are usually catalyzed by the ATP-dependent invertase/hexokinase reaction, but can be replaced by the PPi-consuming sucrose synthase/UDP-glucose pyrophosphorylase reaction. The activation of this alternative pathway of sucrose catabolism upon oxygen deficiency stress was confirmed for several plant species, and the substitution of the ATP-dependent reactions by the UTP and PPi-dependent pair of enzymes is generally accepted (Springer et al., 1986; Ricard et al., 1991, 1998; Guglielminetti et al., 1995; Perata et al., 1996, 1997; Biemelt et al., 1999; Mustroph and Albrecht, 2003; Albrecht et al., 2004; Bailey-Serres and Voesenek, 2008).

The second phosphorylation step in the glycolytic pathway is the phosphorylation of fructose-6-phosphate to fructose-1,6-bisphosphate via phosphofructokinase. The reaction can be performed by an ATP-dependent phosphofructokinase (PFK) or by the PPi-dependent form pyrophosphate-fructose-6-phosphate-phosphotransferase (PFP). PFP consists of two different subunits (PFP-alpha, PFP-beta) that form a heterotetramer (Wong et al., 1990; Teramoto et al., 2000), whereas the subunit composition of a PFK complex is not known yet.

The contribution of each phosphofructokinase enzyme to the phosphorylation step and their functions during different growth conditions remain unclear. While during hypoxic stress both enzyme activities are induced in wheat and maize roots (Mustroph and Albrecht, 2003), their activities do not change in roots of potato plants (Mustroph et al., 2005). Experiments with transgenic potato plants with a severe decrease in PFP activity revealed no change in aerobic (Hajirezaei et al., 1994) or anoxic metabolism (Mustroph, unpublished results). However, the PFP activity was increased in anoxically germinated rice coleoptiles (Mertens et al., 1990; Kato-Noguchi, 2002) and anoxic rice suspension cells (Mohanty et al., 1993), whereas PFK activity was unaffected.

Studies on genes encoding the ATP- and PPi-dependent phosphofructokinases have been previously limited by the lack of information on coding sequences for plant PFKs, which were only recently described (Mustroph et al., 2007; Winkler et al., 2007). The *Arabidopsis thaliana phosphofructokinase* gene family consists of 11 members, of which four members encode PFPs and seven encode PFKs (Nielsen et al., 2004; Mustroph et al., 2007; Winkler et al., 2007). Here, we identify 15 putative *phosphofructokinase* genes of rice, five encoding PFPs and 10 encoding PFKs, and perform transcriptomic and enzymatic studies to evaluate the contribution of *phosphofructokinase* genes to anoxic metabolism in roots, stems, and leaves of the highly anoxia-tolerant plant. The analysis includes evaluation of *phosphofructokinase* gene expression during darkness and illumination, since it was already demonstrated that illumination greatly enhances the tolerance of plants to anoxic stress due to the contribution of photosynthesis to energy production (Mustroph et al., 2006b). The findings indicate that individual members of the *PFK* and *PFP* gene families are induced by anoxia, although activities of these two enzymes are only slightly increased by the stress. Notably, the induction of *PFP* genes was greater in the shoots and leaves than in roots, leading to the suggestion that the induction of the PPi-utilizing phosphofructokinase may only occur in tissues with sufficient carbohydrate levels for consumption during the stress. During the study, mRNA sequences and intracellular localization of the proteins were analyzed, and several discrepancies to the annotated versions were found.

## Materials and Methods

### Plant material and anoxic treatment

Rice seeds (*Oryza sativa* ssp. *indica* cv. Cigalon) were watered, germinated for 3 days in the dark at 27°C and transferred to pots containing Knop nutrient solution that was continuously aerated (Mustroph and Albrecht, 2003). After growth for 20 days in 16 h-light/8 h-dark cycles and 250 μmoles photons m^−2^ s^−1^ the plants were placed in desiccators, while their roots were immersed in nutrient solution. The gaseous and the aqueous phase (the nutrient solution) in the desiccator were continuously flushed with nitrogen gas and plants were treated for 0.5, 2, 8, or 24 h in the light (250 μmoles photons m^−2^ s^−1^) or in complete darkness. Additionally, light-grown plants were exposed to 24 h darkness in ambient air to distinguish between light-dependent and anoxia-independent gene expression regulation. For harvest, plants were removed from the desiccator, divided into roots, stem and leaf sheaths, and frozen immediately in liquid nitrogen within 60 s.

### Isolation of RNA, cDNA synthesis, and semi-quantitative reverse-transcriptase polymerase chain reaction analysis

RNA from frozen tissues was extracted using the Trizol reagent (Bioline GmbH, Luckenwalde, Germany). cDNA was synthesized from 15 μg RNA with standard protocols using oligodT primers and MLV reverse transcriptase (Fermentas GmbH, St. Leon-Rot, Germany). The PCR reactions were performed using Taq polymerase (New England Biolabs GmbH, Frankfurt, Germany) according to the purchaser’s protocol. The oligonucleotide primers used are listed in Table S1 in Supplementary Material. Because of the high GC content of rice cDNA, for each primer combination the optimal PCR conditions were tested using different concentrations of MgCl_2_, DMSO, betaine, cycle numbers, and temperatures. The optimal conditions for the semi-quantitative amplification of each fragment from cDNA are summarized in Table S2 in Supplementary Material. PCR products were analyzed by electrophoresis in standard 1% agarose gels. DNA bands were quantified by use of the program AlphaEaseFC (Alpha Innotech Corporation).

### Cloning and sequencing of PFKs and PFPs

For intracellular localization of the proteins and confirmation of nucleotide sequences, full-length *PFKs* were amplified from cDNA with VELOCITY DNA polymerase (Bioline, Germany) by use of the primers listed in Table S1 in Supplementary Material and cloned into the vector pDONR221 by use of the Gateway technology (Invitrogen, Germany). The resulting entry clones were completely sequenced by Sanger sequencing and compared to the reference sequences from the rice genome annotation project (Ouyang et al., 2007)^1^. Subsequently, correct clones were transferred via the LR reaction into the vector pEarleyGate 103 (Earley et al., 2006), which had been modified by the addition of one base in order to put the C-terminal GFP into frame. The plasmids were sequenced again to verify the correct frame of the PFK-GFP fusion.

The two sequences *OsPFK07* and *OsPFK08* did not fully match the annotated mRNA sequences. In this case, several clones and fragments of cDNA as well as of genomic DNA were sequenced, not only from the variety Cigalon, but also from other varieties (Nipponbare, M202, FR13A, CT6241, Dongjin, Hwayoung). For *OsPFK07*, a new full-length construct was made based on the truncated version of the Cigalon variety.

After the first localization studies and the observation of aggregate formation in transiently transformed tobacco leaves, we also cloned the N-terminal ca. 100 amino acids to obtain truncated *PFK* sequences by use of the primers listed in Table S1 in Supplementary Material. Here, products were amplified with Phusion DNA Polymerase (Fermentas GmbH, St. Leon-Rot, Germany), and cloned by the Gateway technology into the vector pDONR221 (Invitrogen, Germany). After sequencing, correct sequences were subcloned by the LR clonase into the vector pK7FWG2,0 (Karimi et al., 2002).

For sequencing of *PFPs*, a major piece of each mRNA sequence was amplified by PCR from cDNA by use of the primers listed in Table S1 in Supplementary Material. The PCR products were directly sequenced and compared to the annotated sequences. Nucleotide sequences differing from the annotated versions were submitted to GenBank with the IDs KC620557-KC620559.

### Transient transformation of tobacco leaves and intracellular localization of PFKs

Binary expression vectors containing PFK-GFP fusion constructs were transformed into the Agrobacteria strains LBA4404 (full-length constructs) and GV3103 (N-terminal truncations). Tobacco leaves were transiently transformed by infiltration with Agrobacteria suspensions as described in Bendahmane et al. (2000) and Mustroph et al. (2007). About 3–4 days after infiltration, leaf disks were collected and protoplasts were isolated as previously described (Bayley et al., 1992; Mustroph et al., 2007). GFP fluorescence was analyzed on either protoplasts or undigested leaf disks by confocal laser scanning microscopy using Leica TCS SP2 (Leica, Germany, at λ_ex_ 488 nm, λ_em_ 530–555 nm for GFP and 650–720 nm for chlorophyll emission).

### Enzyme activities

Plant tissue was ground in liquid nitrogen to a fine powder and extracted in 50 mM Hepes-KOH, pH 6.8 containing 5 mM Mg acetate, 5 mM β-mercaptoethanol, 15% (v/v) glycerol, 1 mM EDTA, 1 mM EGTA, 5 mM DTT, and 0.1 mM Pefabloc proteinase inhibitor (Boehringer Mannheim, Germany). The homogenate was centrifuged at 13,000 *g* at 4°C for 15 min. The resulting supernatant was used for spectrophotometric determination of PFK and PFP activities as well as the fermentative enzymes alcohol dehydrogenase (ADH) and pyruvate decarboxylase (PDC) at 340 nm using a UVIKON photometer (Kontron, Germany).

For the assay of PFK (EC 2.7.1.11), the reaction mixture was 0.1 M Hepes-KOH, pH 7.9 with 2 mM MgCl_2_, 0.15 mM NADH, 7.5 mM fructose-6-phosphate, 1 U aldolase, 1 U triosephosphate isomerase, and 1 U glycerol-3-phosphate-dehydrogenase (Sigma-Aldrich, Germany). The reaction was started by addition of 2.5 mM ATP. For assay of PFP (EC 2.7.1.90), the same reaction mixture was used with the addition of 1 μM fructose-2,6-bisphosphate, and initiation of the reaction with 1 mM NaPPi (modified from Gibbs et al., 2000). PDC (EC 4.1.1.1) was assayed in 50 mM MES, pH 6.8, with 25 mM NaCl, 1 mM MgCl_2_, 0.5 mM thiamine pyrophosphate, 2 mM dithiothreitol, 0.17 mM NADH, 50 mM sodium oxamate, 10 U ADH (Sigma-Aldrich, Germany) and the reaction was initiated by addition of 10 mM pyruvate (Waters et al., 1991). ADH (EC 1.1.1.1) was assayed in 50 mM TES buffer, pH 7.5, with 0.2 mM NADH and the reaction was initiated by the addition of 10 mM acetaldehyde (Waters et al., 1991). Protein concentration was measured according to Bradford (1976).

### Bioinformatics and statistics

Nucleotide and protein sequences were analyzed by use of the program Bioedit (Tom Hall, Ibis Biosciences, Carlsbad, USA). ClustalW alignments were done with the website http://www.genome.jp/tools/clustalw/. Phylogenetic trees were constructed by use of the website http://www.phylogeny.fr/version2_cgi/phylogeny.cgi. Expression data from rice microarray experiments were obtained from Mustroph et al. (2010), and heat maps were drawn with TIGR-MeV (Saeed et al., 2006).

Polymerase chain reaction analyses were performed on three independent bioreplicates. Infiltration of tobacco leaves was repeated three to five times for each construct. Enzymatic measurements were done with three independent bioreplicates, and results were statistically analyzed with the package “multcomp” in R by use of the Tukey HSD test.

## Results

### Identification of genes encoding phosphofructokinases in rice and comparison of their sequences

**Figure 1:**
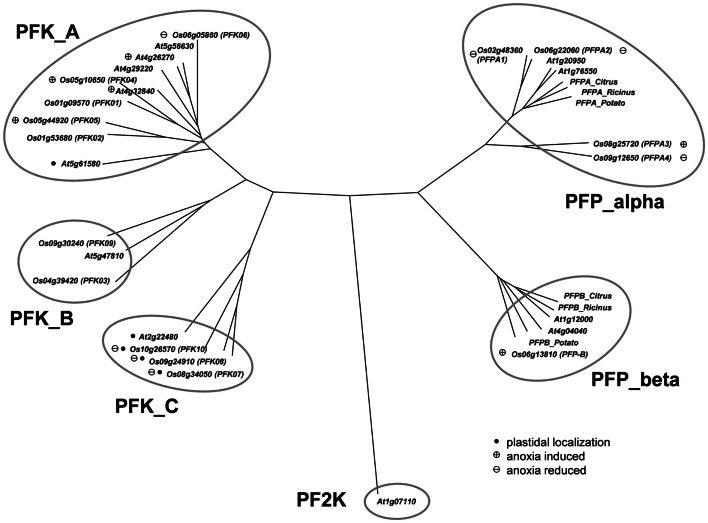
**Phylogeny of plant phosphofructokinase genes**. Unrooted neighbor joining tree produced from CLUSTALW alignment of amino acid sequences of *Arabidopsis* and rice *phosphofructokinase* genes. Genes marked with a “plus” showed increased transcript levels and names marked with a “minus” showed decreased transcript levels during anoxic stress treatment, respectively. *Arabidopsis* genes marked with a plus are those that were induced by >2-fold in published microarray experiments (Mustroph et al., 2010). Genes marked with a black dot are plastid localized. PF2K: *Arabidopsis* *phosphofructo-2-kinase* gene (At1g07110) used as an outgroup in the phylogenetic analysis. Accession numbers for other plant *PFPs*: Citrus_Alpha, AAC67587; Potato_Alpha, P21342; Ricinus_Alpha, Q41140; Citrus_Beta, AAC67586; Potato_Beta, P21343; Ricinus_Beta, Q41141.

The *Arabidopsis* phosphofructokinase-encoding gene sequences were used to perform a BLAST-search of the rice genome (Ouyang et al., 2007)^2^ for homologous nucleotide sequences. This analysis identified 15 different sequences with high similarity to *Arabidopsis* phosphofructokinase-encoding genes. A phylogenetic tree based on the amino acid sequences was generated, including the sequence of phosphofructo-2-kinase/fructose-2,6-bisphosphatase, which is a similar, but unrelated protein (Figure 1**)**. Four of the rice proteins (LOC_Os02g48360, LOC_Os06g22060, LOC_Os08g25720, LOC_Os09g12650) grouped with the two putative *Arabidopsis* PFP-alpha subunits (At1g20950, At1g76550) and known PFP-alpha subunits from other plants. Surprisingly, two rice PFP-alpha protein sequences (LOC_Os08g25720, LOC_Os09g12650) were found to be distinct from the PFP-alpha subunit sequences of dicotyledonous species known so far (Figure 1). One rice amino acid sequence (LOC_Os06g13810) showed high homology to the two putative *Arabidopsis* PFP-beta subunits (At1g12000, At4g04040) and the PFP-beta subunits from *Citrus* × *paradisii*, potato and *Ricinus communis*.

The remaining 10 rice phosphofructokinases grouped with seven *Arabidopsis* PFK proteins, indicating that both species have larger families of ATP-dependent than PPi-dependent phosphofructokinases. Based on the sequence similarities among the PFK family, three sub-clades were distinguished (Figure 1). One group includes five rice (LOC_Os01g09570, LOC_Os01g53680, LOC_Os05g10650, LOC_Os05g44920, LOC_Os06g05860) and five *Arabidopsis* PFKs, the two other groups have each one *Arabidopsis* member (At2g22480, At5g47810), and two (LOC_Os04g39420, LOC_Os09g30240), or three (LOC_Os08g34050, LOC_Os09g24910, LOC_Os10g26570) rice members, respectively (Figure 1). Based on these results, we propose to distinguish the three PFK sub-clades as PFK_A, PFK_B, and PFK_C (Table 1). The amino acid alignment reveals distinct sequence patterns with high similarity among the members of each sub-clade that differentiate them from the other two sub-clades (Figure S1 in Supplementary Material). Besides other sequence differences, members of group B have shorter N- and C-termini, whereas members of group C have shorter C-termini compared to group A members. Furthermore, all members of group C are predicted to be localized to plastids (Table 1; TargetP, Emanuelsson et al., 2000; pSort, Horton et al., 2006).

**Table 1:** **Rice *phosphofructokinase* genes, Locus identifier, length of coding sequence (number of bases), and protein size (number of amino acids)**.

Locus ID	Name	Length of mRNA	Length of protein	Predicted localization	RNAseq expression level from 16 tissues	
					Maximum	Mean
**PFK_A**						
LOC_Os01g09570	*OsPFK01*	1596	531	Cytosolic	69.04	27.60
LOC_Os01g53680	*OsPFK02*	1683	560	Cytosolic	44.50	10.24
LOC_Os05g10650	*OsPFK04*	1629	542	Cytosolic	2.89	1.40
LOC_Os05g44922	*OsPFK05*	1704	567	Cytosolic	53.36	14.39
LOC_Os06g05860	*OsPFK06*	1677	558	Plastid (confirmed cytosolic)	118.48	33.60
**PFK_B**						
LOC_Os04g39420	*OsPFK03*	801	266	Cytosolic	46.80	6.89
LOC_Os09g30240	*OsPFK09*	1398	465	Cytosolic	48.42	5.79
**PFK_C**						
LOC_Os08g34050	*OsPFK07*	1590 (1608)	529 (535)	Plastid	7.98	2.10
LOC_Os09g24910	*OsPFK08*	1584 (1665)	527 (555)	Plastid	14.83	4.51
LOC_Os10g26570	*OsPFK10*	1575	524	Plastid	41.14	6.07
**PFP-ALPHA**						
LOC_Os02g48360	*OsPFPA1*	1854	617	Cytosolic	98.49	19.19
LOC_Os06g22060	*OsPFPA2*	1869	622	Cytosolic	171.15	53.64
LOC_Os08g25720	*OsPFPA3*	1854	617	Cytosolic	102.03	42.51
LOC_Os09g12650	*OsPFPA4*	1884 (1722)	627 (573)	Cytosolic	0.68	0.36
**PFP-BETA**						
LOC_Os06g13810	*OsPFP-B*	1704	567	Cytosolic	110.02	52.99

*Numbers for *OsPFK07* are for the sequence of the variety FR13A. Numbers in brackets are annotated sizes from the rice genome annotation project (http://rice.plantbiology.msu.edu; Ouyang et al., 2007). Intracellular localization was predicted by use of two online programs (TargetP, Emanuelsson et al., 2000; pSort, Horton et al., 2006). RNAseq expression data are from the Nipponbare variety from 16 tissues, summarized from the rice genome annotation project (http://rice.plantbiology.msu.edu/expression.shtml)*.

### Expression of phosphofructokinase genes under aeration

**Figure 2:**
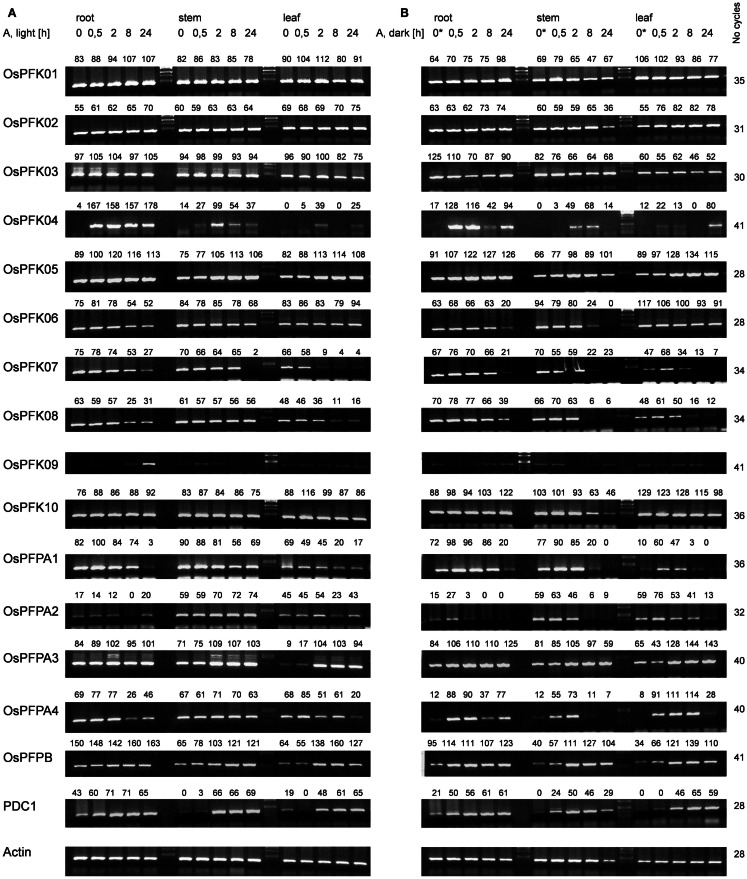
**Semi-quantitative RT-PCR analysis of transcript abundance of the rice *phosphofructokinase* genes**. Total RNA was extracted from roots, stems, and leaves of 3-week-old rice plants under normoxia or after different durations of anoxic stress. Samples in **(A)** were treated in the light; samples in **(B)** were treated in the dark. The sample 0* was harvested after 24 h in darkness under normoxic conditions. *Actin* and *PDC1* (Os05g39310) transcript levels were used to control for equal loading and to confirm low-oxygen stress, respectively. RT-PCR product levels were quantified by use of the program AlphaEaseFC (Alpha Innotech Corporation), and normalized to the level of actin mRNA in the same sample. The relative transcript level is indicated above each band (% of light control). Gel images are representative of three independent biological replicate experiments. RT-PCR conditions are listed in Table S2 in Supplementary Material.

To study the expression patterns of all representatives of the rice *phosphofructokinase* gene family, transcript abundance was analyzed by use of semi-quantitative reverse transcription polymerase chain reaction (RT-PCR) using total RNA from roots, leaf sheaths, and stems of rice seedlings grown under non-stress conditions in the light or after transfer to complete darkness for 24 h (labeled as 0 h* in Figure 2). The abundance of *actin* mRNA was used as a loading control (Ren et al., 2005). The accumulation of some transcripts encoding PFKs and PFPs were organ-specifically and/or light-dependently regulated, while others exhibited ubiquitous expression patterns. mRNAs for *OsPFK01 – OsPFK03*, *OsPFK05 – OsPFK08*, and *OsPFK10* were detected in all analyzed organs of seedlings exposed to light, as well as transcripts of *OsPFPA1* and *OsPFPA4* (Figure 2). *OsPFK04* and *OsPFK09* mRNAs were present at low levels in tissues of non-stressed seedlings during both light and dark exposure (0 h anoxia). *OsPFPA2* was strongly expressed in the stem, and *OsPFPA3* as well as *OsPFP-B* showed lower expression in leaves than in roots and stems. The transfer of seedlings to darkness for 24 h resulted in a decrease in transcripts encoding *OsPFPA4* in all tissues, and *OsPFK07* and *OsPFPA1* in leaves (0 h* in Figure 2). *OsPFPA3* transcript levels were slightly enhanced during darkness, whereas transcripts of *OsPFK01 – OsPFK03, OsPFK05, OsPFK06, OsPFK08, OsPFK10, OsPFKPFPA2*, and *OsPFKPFP-B* accumulated to similar extent during dark and light growth.

### Expression of phosphofructokinase genes under anoxia in light and darkness

To identify the *phosphofructokinase* genes that may play a tissue specific role in the response to anoxic metabolism in rice seedlings, transcript levels of the 10 *PFK* and 5 *PFP* genes were quantified in seedling tissues following exposure to anoxia (30 min to 24 h), in the light or complete darkness. The *pyruvate decarboxylase* (*PDC1, LOC_Os05g39310*) transcript, which is known to significantly increase in abundance under low-oxygen conditions in rice seedlings, was monitored as positive control for anoxic stress (Figure 2). Under normoxic conditions, the *PDC1* mRNA was more abundant in roots as compared to stems and leaves. *PDC1* transcript level increased within 30 min of anoxia stress in roots, whereas the increase in stems and leaves was detected after 2 h of stress. *PDC1* transcripts were induced by anoxia in roots and leaves with similar kinetics in seedlings exposed to light or darkness. In stems, the induction of *PDC* was slightly faster in darkness than during light irradiation.

The evaluation of anoxic stress-responsive mRNA accumulation of the rice *PFK* and *PFP* genes identified three groups. Expression of group 1 *phosphofructokinase* genes (*OsPFK01, OsPFK02*, *OsPFK03*) did not change during stress treatment (Figure 2). Group 2 *phosphofructokinase* genes (*OsPFK06, OsPFK07, OsPFK08, OsPFK10, OsPFPA1, OsPFPA2, OsPFPA4*) showed a decrease in transcript levels in response to anoxia. Among these genes, *OsPFK10* and *OsPFPA2* transcripts were reduced in stems only after 24 h anoxia in darkness, whereas the transcript abundance of the other five genes reduced more rapidly. *OsPFK06* transcripts decreased in roots and stems, whereas *OsPFK07, OsPFK08, OsPFPA1*, and *OsPFPA4* transcripts decreased in all analyzed tissues. Interestingly, all *PFK* genes in group 2 that showed reduced expression under anoxia are predicted to be plastid-localized (Table 1).

*Phosphofructokinase* genes of group 3 were induced under anoxia (*OsPFK04, OsPFK05, OsPFPA3, OsPFP-B*). *OsPFK09* is also included in group 3, but displayed a markedly delayed increase in mRNA accumulation in response to stress at an extremely low expression level. *OsPFK04*, predicted to encode a cytosolic enzyme, expresses the strongest anoxia-induced transcript of group 3. *OsPFK04* mRNA content was below the detection level in seedlings grown under aerated conditions, but increased dramatically within 30 min of anoxia in roots resembling the pattern of *PDC1* accumulation. *OsPFK05* mRNA accumulated less dramatically only in leaves after 2–24 h of anoxia. Among the *PFP* genes, only the content of *OsPFPA3* and *OsPFP-B* mRNAs slightly increased in leaves and stems after 2 h of anoxia (Figure 2), resembling the expression of *OsPFK05*. The group 2 and group 3 *phosphofructokinase* genes are highlighted in the phylogenetic tree with a minus or plus sign, respectively, for their low-oxygen stress responsiveness (Figure 1).

Exposure to light or darkness most dramatically altered mRNA content of group 2 genes of the *phosphofructokinase* family. These genes showed a higher decrease in transcript levels in response to anoxia in darkness as compared to anoxia during illumination. *OsPFPA1* and *OsPFPA4* mRNA content was reduced during dark anoxia most likely in response to the transfer to darkness, as 24 h dark incubation under constant aeration (sample 0 h* in Figure 2) caused a similar loss of the mRNA level. On the other hand, *OsPFK06, OsPFK07, OsPFK08, OsPFK10*, and *OsPFPA2* transcript levels were decreased stronger during anoxia in darkness than in light, but not during darkness alone.

### Sequence variations of phosphofructokinase genes

**Figure 3:**
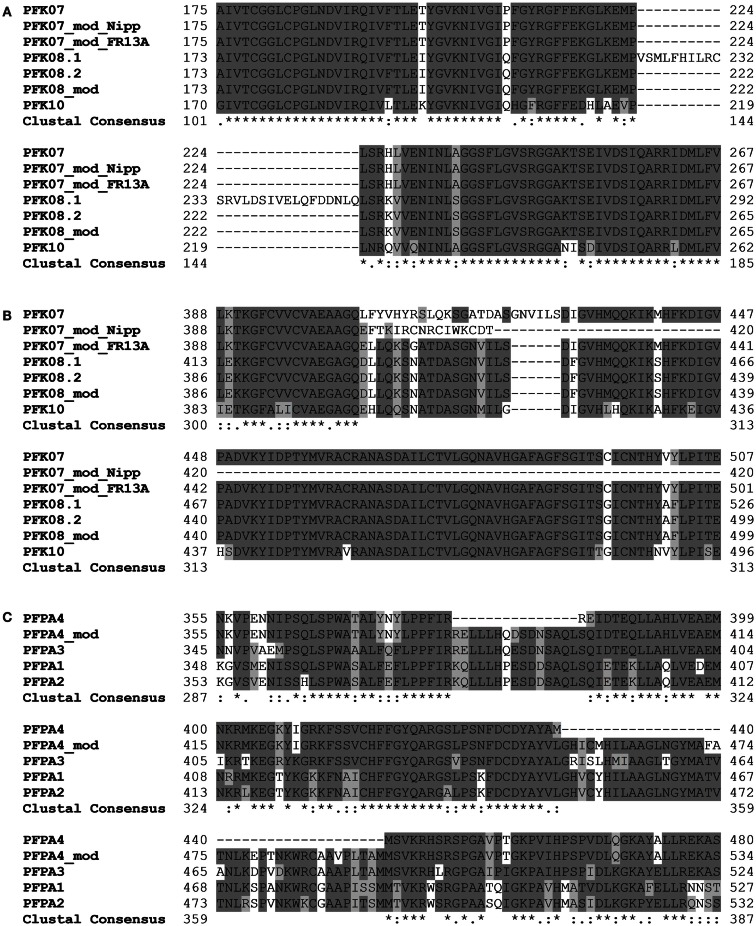
**Sections of protein alignments for different phosphofructokinase subgroups, whose sequences differ from the annotated public sequences (http://rice.plantbiology.msu.edu, Ouyang et al., 2007)**. New sequences that were confirmed by sequencing of PCR products are indicated as “modified” (mod). Full-length nucleotide and protein sequences can be found in Figure S2 in Supplementary Material. **(A)** Two splicing forms of *OsPFK08*; **(B)** modified splicing for *OsPFK07*, leading to a frame shift in the protein, and the modified sequence in the rice variety FR13A; **(C)** modified splicing of *OsPFPA4*.

Amplification and sequencing of *PFK* genes revealed in the *OsPFK07* and *OsPFK08* sequences a significant difference to annotated sequences. *OsPFK08* was annotated with two splicing forms (Ouyang et al., 2007)^3^. The longer form *LOC_Os09g24910.1* contained a sequence that was not present in any other sequence of the group PFK_C (Figure 3A; Figures S1 and S2 in Supplementary Material). Sequencing of several cDNA clones derived from RNA of different rice varieties revealed the unique presence of the shorter annotated splicing form, *LOC_Os09g24910.2* (Figure 3A and data not shown). *OsPFK07* surprisingly revealed the lack of 20 nucleotides in its mRNA sequence in comparison to the annotated version, leading to a frame shift in the translated protein sequence and a premature stop codon (Figure 3B). We re-sequenced the *OsPFK07* cDNA and genomic DNA of several rice varieties in order to confirm the initial findings. Genomic clones of *OsPFK07* of all varieties consistently resulted in the complete annotated sequence including the 20 nucleotide region, while sequencing of the *OsPFK07* cDNA of these varieties gave always rise to a truncated sequence lacking this stretch of 20 nucleotides. As result, a truncated protein lacking 120 amino acids at the C-terminus is predicted to be most likely non-functional. Supposedly, this mutation could occur in the rice genome since two other members of the PFK_C are available, while *Arabidopsis* only contains one isoform of this subgroup (Figure 1). In consistency with a putative non-functional gene, the expression level of *OsPFK07* is very low in many tissues, as derived for example from RNAseq analyses (Table 1). However, this finding was not confirmed in our experiments (Figure 2).

Interestingly, two rice varieties, FR13A and CT6241, revealed a second modification of the *OsPFK07* gene, an insertion of two bases in the genomic sequence close to the modified splicing site (Figure S2 in Supplementary Material). This altered sequence restores the reading frame comparable to *OsPFK08* and *OsPFK10* (Figure 3B). However, which of the two sequence variants evolved earlier during evolution remains to be explored in future.

Our sequencing revealed also a modified *OsPFPA4* nucleotide sequence. The annotated sequence lacks part of the final coding sequence leading to encoded PFP-alpha subunit with a truncated C-terminus in comparison to the other three isoforms. However, the sequencing results revealed another mRNA sequence that represents an un-annotated alternative splicing form, which more closely resembles the sequence of the other three homologous genes (Figure 3C; Figure S2 in Supplementary Material). Interestingly, the expression level of *OsPFPA4* was very low in rice tissues as observed by RNAseq analyses (Table 1), but in our analyses *OsPFPA4* was significantly expressed in detectable amounts in seedlings (Figure 2).

### Intracellular localization of PFKs

**Figure 4:**
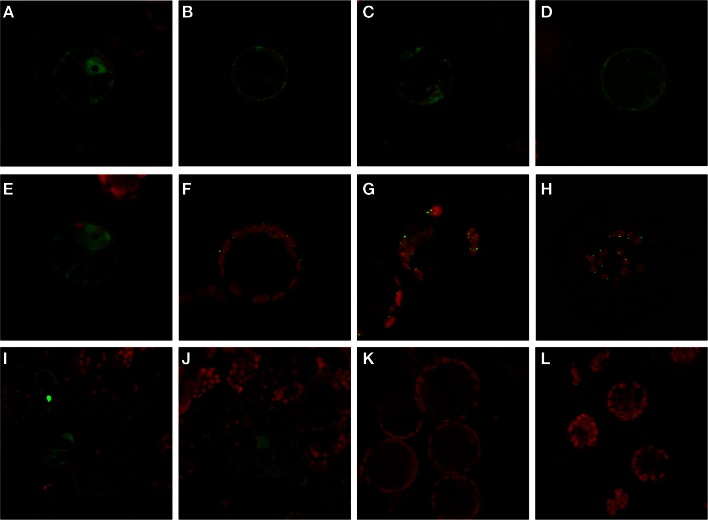
**Subcellular localization of PFK isoforms from rice**. Full-length mRNA sequences or the N-terminal part of the sequences were cloned in frame in front of a GFP-coding sequence and were transiently transformed into tobacco leaves by Agrobacterium infiltration. After 3–4 days, protoplasts or undigested leaf disks were isolated and analyzed by confocal microscopy. Green color represents GFP fluorescence, red color represents chlorophyll autofluorescence. N-terminal part: **(A)** OsPFK01; **(B)** OsPFK02; **(C)** OsPFK04; **(D)** OsPFK05; **(E)** OsPFK06; **(K)** OsPFK08, **(L)** OsPFK10. Full-length CDS: **(F)** OsPFK07; **(G)** OsPFK08; **(H)** OsPFK10; **(I)** OsPFK03; **(J)** OsPFK09.

The expression analysis under anoxia revealed decreased expression of several *PFK* genes, in particular those representatives that encode putative plastid-localized proteins. We therefore aimed to confirm the intracellular localization of PFK isoforms, and applied a similar approach as previously shown for *Arabidopsis* PFKs (Mustroph et al., 2007). Using the leaf infiltration technique for transient transformation of the rice *PFK* genes in tobacco leaves, we showed the clear localization of 5 out of 10 different PFK isoforms in the cytoplasm. Expression of the full-length PFKs of *OsPFK01, OsPFK02, OsPFK04, OsPFK05*, and *OsPFK06* resulted in their cytosolic localization (Figure S3 in Supplementary Material), but the proteins formed cytoplasmic aggregates. Several modified protocols of transient transformation were tested, but resulted always in the production of aggregates: changes in incubation time of transiently transformed tobacco leaves, at different temperatures, use of different Agrobacteria strains and binary vectors, and ultimately stable transformation in *Arabidopsis* (data not shown). However, expression of fusion proteins containing the first 100 amino acids of the respective PFK protein resulted in even distribution throughout the cytosol (Figure 4). Surprisingly, *OsPFK06*, which was predicted to be plastid-localized, was clearly cytosolic (Figure 4E). As a very short transit peptide of only 10 amino acids was predicted (TargetP, Emanuelsson et al., 2000), it is suggested that the organellar localization is a false prediction.

*OsPFK03* and *OsPFK09* are both members of the weakly expressed PFK_B subgroup and are mainly expressed in seeds (Figure S4 in Supplementary Material). Tobacco leaves infiltrated with the truncated or full-length constructs p35S::OsPFK03-GFP and p35S::OsPFK09-GFP did surprisingly not show GFP fluorescence in tobacco mesophyll cells in four independent experiments, although the same vectors, bacterial strains and conditions had been used as for the positive control p35S::OsPFK05-GFP. However, we observed GFP fluorescence in a few epidermal cells that demonstrate cytosolic localization of the proteins (Figure 4; Figure S3 in Supplementary Material). It can be concluded that both proteins are less stable during transient overexpression in tobacco leaves than members of the PFK_A group.

All members of the PFK_C group, *OsPFK07, OsPFK08*, and *OsPFK10*, were predicted to be localized in plastids, with a predicted transit peptide consisting of 30–50 amino acid residues. Our assay revealed localization of the full-length proteins in plastids, however associated with the formation of aggregates (Figure 4), as previously described for *Arabidopsis AtPFK4* and *AtPFK5* (Mustroph et al., 2007). Still, we observed clear association of the aggregates with chloroplasts, and not with other compartments (Figure 4; Figures S3S,T in Supplementary Material). Expression of the N-terminal part did not result in detectable plastidal GFP fluorescence (Figures 4K,L).

### Phylogenetic analysis and expression under oxygen deficiency stress

**Figure 5:**
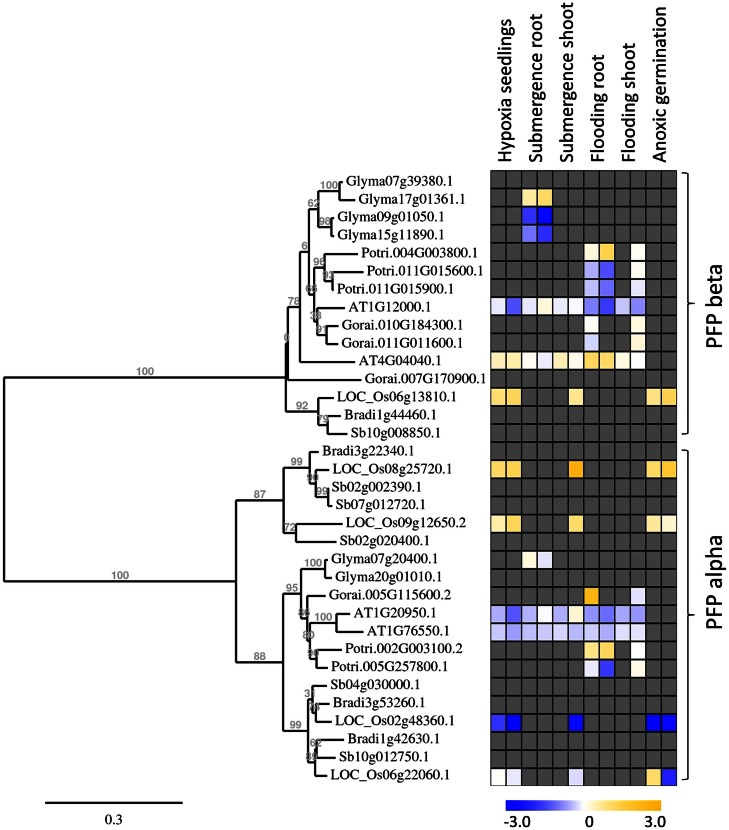
**Phylogenetic tree of plant PFP genes, constructed by the platform http://www.phylogeny.fr (Dereeper et al., 2008)**. Protein sequences were obtained from Phytozome, with modifications after sequencing of rice genes. Gene expression data were collected into a heat map, when available. Data are from the following sources: Hypoxia seedlings: *Arabidopsis* (Branco-Price et al., 2008, 2 and 9 h), rice (Narsai et al., 2009, 3 and 6 h); Submergence root/shoot: *Arabidopsis* (Lee et al., 2011, 7 and 24 h), rice (Mustroph et al., 2010, 24 h shoot); soybean (Nanjo et al., 2011, 6 and 12 h, roots only); Flooding root/shoot: *Arabidopsis* (Hsu et al., 2011, 3 and 12 h), cotton (Christianson et al., 2010, 4 h root and 24 h shoot), poplar (Kreuzwieser et al., 2009, 5 and 24 h root, 168 h shoot); Anoxic germination: rice (Narsai et al., 2009, 24 h; Lasanthi-Kudahettige et al., 2007, 96 h).

**Figure 6:**
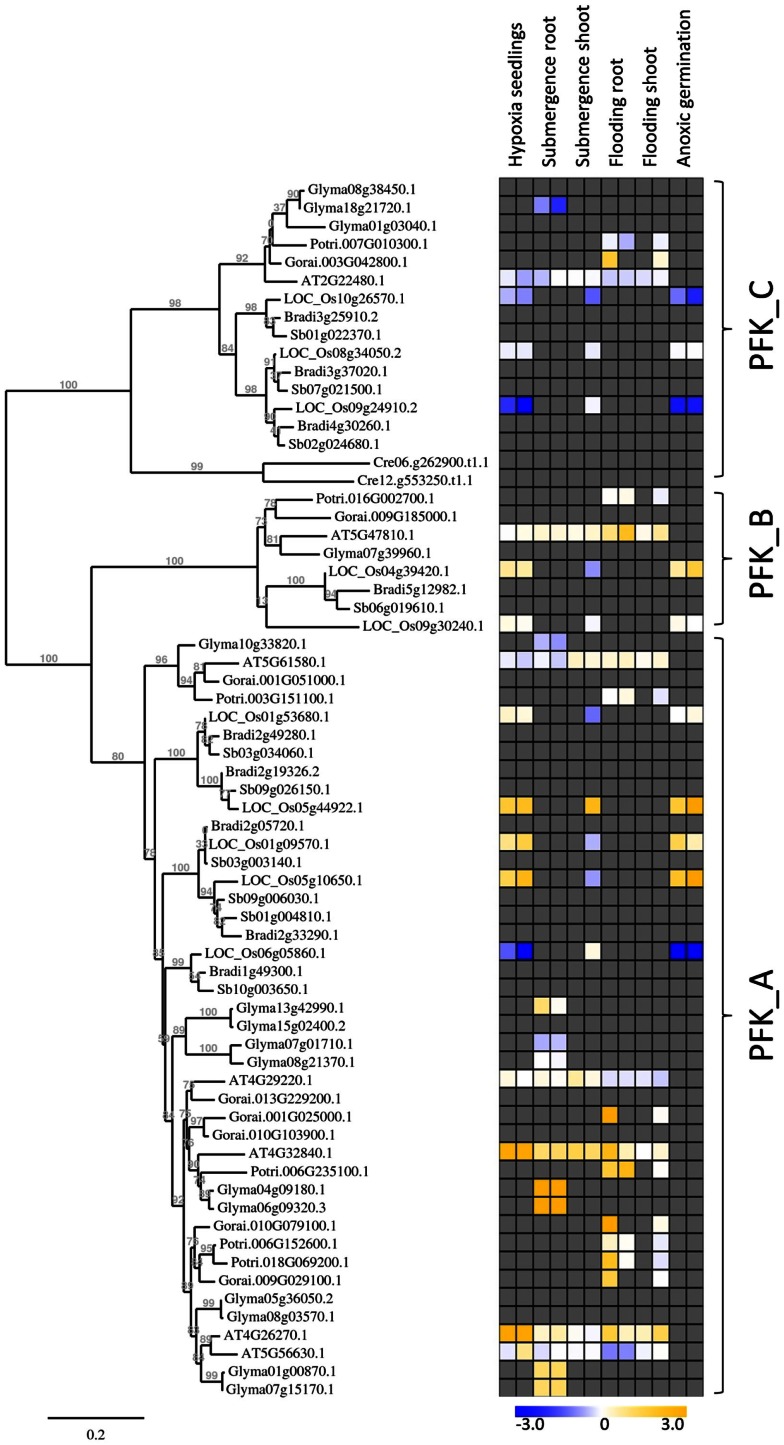
**Phylogenetic tree of plant *PFK* genes, constructed by the platform http://www.phylogeny.fr (Dereeper et al., 2008)**. Protein sequences were obtained from Phytozome, with modifications after sequencing of rice genes. *Chlamydomonas reinhardtii phosphofructokinase* genes were added for comparison. Gene expression data were collected into a heat map, when available. Data are from the following sources: Hypoxia seedlings: *Arabidopsis* (Branco-Price et al., 2008, 2 and 9 h), rice (Narsai et al., 2009, 3 and 6 h); Submergence root/shoot: *Arabidopsis* (Lee et al., 2011, 7 and 24 h), rice (Mustroph et al., 2010, 24 h shoot); soybean (Nanjo et al., 2011, 6 and 12 h, roots only); Flooding root/shoot: *Arabidopsis* (Hsu et al., 2011, 3 and 12 h), cotton (Christianson et al., 2010, 4 h root and 24 h shoot), poplar (Kreuzwieser et al., 2009, 5 and 24 h root, 168 h shoot); Anoxic germination: rice (Narsai et al., 2009, 24 h; Lasanthi-Kudahettige et al., 2007, 96 h).

The expression analysis of *phosphofructokinase* genes of rice under anoxia revealed differential gene expression of several members in roots and shoots of seedlings. For a wider comparison among species, we first identified *phosphofructokinase* genes in different plant species by use of the Phytozome genome collection^4^, and constructed a separate phylogenetic tree for *PFK* and *PFP* sequences (Figures 5 and 6). We selected *phosphofructokinase* genes from plant genomes, of which expression data are available under oxygen deficiency stress, and additionally used two other monocotyledonous species, *Sorghum bicolor* and *Brachypodium distachyon*. Comparison of the genome of the green algae *Chlamydomonas reinhardtii* revealed the presence of *PFK_C* genes only, hinting at a complex evolution of *phosphofructokinase* genes in higher plants. Gene expression data from published microarray experiments were added from rice (Lasanthi-Kudahettige et al., 2007; Narsai et al., 2009; Mustroph et al., 2010), *Arabidopsis* (Branco-Price et al., 2008; Hsu et al., 2011; Lee et al., 2011), poplar (Kreuzwieser et al., 2009), cotton (Christianson et al., 2010), and soybean (Nanjo et al., 2011). It was intended to obtain a first qualified overview about the potential role of phosphofructokinases under oxygen deficiency stress.

Interestingly, all analyzed monocotyledonous species only contained one gene for the beta-subunit of PFP, while dicotyledonous species usually contained more isoforms. In general, PFP-beta-encoding genes of either monocotyledonous or dicotyledonous species show only a low induction upon low-oxygen stress, among them the rice *PFP-B*-encoding gene being up to 3.8 times induced in the microarray experiments (Table S3 in Supplementary Material). This induced expression was shown to a similar extent in our experiments (Figure 2). Transcript levels of three PFP-alpha subunit-encoding genes were reduced or unchanged under oxygen deficiency stress in rice, while *OsPFPA3* was moderately induced in leaves, as indicated by RT-PCR (Figure 2) and microarray analyses (Figure 5). This latter gene was grouped into a monocotyledonous-specific sub-clade of PFP-alpha subunit encoding sequences. The genes most similar to dicotyledonous species were down-regulated, similar to the PFP-alpha subunit encoding genes of other plant species, whose expression was hardly induced (Figure 5). It would be interesting to analyze if other members of this monocotyledonous-specific subgroup are also induced by oxygen deficiency stress, for example in *Sorghum* or *Brachypodium*, or if this induction is specific to the submergence-tolerant rice plant.

Among the *PFK* gene family, each plant species analyzed contained members that were induced by oxygen deficiency stress (Figure 6), indicating an important function in plant metabolism under the stress condition. Most of the induced genes, among them *OsPFK04, OsPFK05, AtPFK3*, and *AtPFK6*, belong to the subgroup PFK_A. The plastidal genes of subgroup PFK_C were generally down-regulated in rice, *Arabidopsis* and soybean (Figure 6; Table S3 in Supplementary Material). *OsPFK06* shows the strongest reduced mRNA content and its encoded PFK isoform is localized in the cytosol (Figure 4E). *OsPFK06* was grouped with two other genes from monocotyledonous species, whose expression under oxygen deficiency stress remains to be determined.

### Activity of phosphofructokinases after anoxic treatment

To further evaluate the relative importance of PFK and PFP during the transition from aerated to anoxic growth conditions in rice seedlings, we measured the specific activities of these two distinct phosphofructokinases along with the fermentative enzymes ADH and PDC (Table 2). In comparison to anoxically treated plants, the ADH showed the lowest basal activity in leaves in ambient oxygen concentration, whereas PDC activity was low in all tissues. Illumination did not affect the basal ADH and PDC activity, but the anoxic induction of these enzymes was more pronounced in illuminated seedlings than in darkness. As expected, a significantly elevated ADH activity was observed after 24 h of anoxia in all tissues. ADH activity increased threefold to ninefold during the 24-h anoxic treatment. Thereby, the greatest increase of activity was observed in leaves relative to the low basal level in this organ. Also for PDC activity, a significant anoxia-induced elevation was determined after 24 h of anoxia (Table 2).

**Table 2:** **Specific activities of phosphofructokinases (PFK, PFP) and fermentative enzymes (ADH, PDC) (nmol × mg protein^−^^1^ × min^−^^1^) in roots, stems, or leaves of rice seedlings after 4 and 24 h of anoxic stress (A), or under normoxic conditions (C)**.

		**PFK**	**PFP**	**ADH**	**PDC**
**ROOT**					
C	24 h Light	24.12 ± 5.45^a^	94.98 ± 28.74^a^	122.26 ± 30.56^a^	0.00 ± 0.00^a^
C	24 h Dark	20.98 ± 1.32^a^	84.27 ± 22.51^a^	116.90 ± 34.21^a^	0.00 ± 0.00^a^
A	4 h Light	20.70 ± 3.42^a^	110.76 ± 29.55^a^	212.13 ± 25.83^a^	1.12 ± 0.98^a^
A	4 h Dark	23.03 ± 7.13^a^	120.55 ± 39.30^a^	256.62 ± 16.24^a^	0.75 ± 1.30^a^
A	24 h Light	31.33 ± 4.45^a^	102.21 ± 11.92^a^	980.43 ± 152.23^b^	21.95 ± 2.32^b^
A	24 h Dark	20.23 ± 0.99^a^	75.16 ± 12.82^a^	692.15 ± 121.28^c^	14.75 ± 3.26^c^
**STEM**					
C	24 h Light	16.55 ± 1.46^bc^	135.34 ± 9.02^a^	186.27 ± 17.02^a^	1.45 ± 1.87^a^
C	24 h Dark	12.44 ± 1.99^c^	132.30 ± 10.22^a^	183.65 ± 11.22^a^	2.29 ± 0.91^a^
A	4 h Light	23.85 ± 2.72^a^	141.81 ± 12.72^a^	325.01 ± 33.06^b^	5.47 ± 1.17^a^
A	4 h Dark	18.33 ± 2.53^b^	131.33 ± 9.61^a^	207.26 ± 0.86^a^	4.38 ± 1.50^a^
A	24 h Light	20.68 ± 1.09^ab^	143.28 ± 4.50^a^	599.35 ± 20.47^c^	16.38 ± 3.08^b^
A	24 h Dark	17.25 ± 1.73^bc^	142.75 ± 7.48^a^	475.82 ± 52.06^d^	16.63 ± 2.20^b^
**LEAF**					
C	24 h Light	11.23 ± 3.46^ac^	21.52 ± 0.98^a^	26.58 ± 3.83^a^	0.00 ± 0.00^a^
C	24 h Dark	5.28 ± 1.25^b^	19.94 ± 1.71^a^	24.69 ± 1.94^a^	0.00 ± 0.00^a^
A	4 h Light	12.54 ± 0.94^ac^	22.16 ± 1.86^a^	75.60 ± 4.48^b^	0.17 ± 0.21^a^
A	4 h Dark	8.58 ± 1.54^bc^	21.39 ± 2.88^a^	55.81 ± 6.78^ab^	0.06 ± 0.11^a^
A	24 h Light	13.74 ± 1.06^a^	25.55 ± 3.09^a^	258.36 ± 31.83^c^	4.10 ± 0.51^b^
A	24 h Dark	8.01 ± 0.90^bc^	23.62 ± 0.21^a^	156.41 ± 7.41^d^	2.76 ± 0.34^c^

*Data are mean values from three biological replicate samples ±SD. Values with the same letter within one organ are significantly different at *P* < 0.05*.

Evaluation of PFK activity revealed a decline of 20 and 50% PFK activity after 24 h dark incubation under normoxia in comparison to the illuminated control in stems and leaves, respectively. By contrast, PFP activity was not modified upon the transfer of seedlings to darkness (Table 2). In general, PFP and PFK activities did only moderately change during anoxic treatment. PFK activity in stems was significantly higher after 4 and 24 h of anoxia in light and in dark anoxic treatment as compared to the respective control (Table 2), which likely results from increased expression of *OsPFK05* in this tissue (Figure 2). PFK activity did not change significantly in response to anoxia in roots or leaves. The activity of PFP was slightly but not significantly increased in leaves only after 24 h of anoxia in light and darkness (Table 2), but not in roots or stems.

## Discussion

### Phosphofructokinase genes in rice and their expression under normoxic conditions

In this report we analyzed the expression of 15 *phosphofructokinase* genes in rice (Table 1). Five gene sequences resemble *PFP* genes derived from other plant species, which have been described earlier (Carlisle et al., 1990; Todd et al., 1995; Kapri et al., 2000; Suzuki et al., 2003). Genes encoding the two different subunits of the heterotetrameric PFP were found in the rice genome (Figure 1). Whereas *Arabidopsis* possesses two genes each encoding the PFP-alpha and PFP-beta subunits (Mustroph et al., 2007), available rice sequence data predicts four genes for PFP-alpha subunits, which represent the regulatory PFP subunit (Table 1), and one gene encoding the PFP-beta subunit (*PFP-B*, *LOC_Os06g13810*) representing the catalytically active subunit of the PFP complex (Yan and Tao, 1984; Theodorou et al., 1992; Theodorou and Plaxton, 1996). It is suggested that rice uses several regulatory subunits with distinct properties that are activated under different growth conditions. Indeed, the four *OsPFPA* genes show diverse expression patterns in plant tissues and under various light conditions (Figure 2). *OsPFPA2* mRNA was detected primarily in stems, whereas *OsPFPA1* and *OsPFPA3* mRNAs were less abundant in leaves than in stems or roots (Figure 2). PFP activity was very low in leaf extracts compared to that of roots and stems (Table 2). Furthermore, *OsPFPA1* and *OsPFPA4* showed dark-dependent decreases in mRNA accumulation in leaves (Figure 2), but the PFP activity after dark incubation was unchanged in comparison to light-grown leaves (Table 2). These observations in rice are consistent with the previous report of decreased abundance of two *Arabidopsis PFP* gene transcripts (At1g12000, At1g20950) after transfer to darkness, although no apparent change in PFP activity was determined (Gibon et al., 2004). We predict that the total *in vitro* enzymatic activity from whole cell extracts conceals the biologically significant differences in the assembly of PFP heterotetrameric complexes in response to the cell-specific variation of the expression of PFP isoforms.

The other 10 *phosphofructokinase* genes (Table 1) showed homology to the seven *AtPFK* genes (Mustroph et al., 2007) (Figure 1). Five of these proteins form a sub-clade, designated the PFK_A group, together with the five AtPFK proteins that are important for cytosolic glycolysis (Figure 1). The present study confirms that four of these rice genes are highly expressed in all observed tissues, with the exception of *OsPFK04* (Figure 2). Interestingly, the rice genome does not contain a true plastidal isoform in this subgroup, since the predicted plastidal *OsPFK06* of the PFK_A group was clearly cytosolic (Figure 4E), while AtPFK4 presents a plastidal isoform in the PFK_A group. The rice PFK_C group members apparently fulfill the sole plastidal PFK function (see below).

The protein sequences of the PFK_B and PFK_C groups markedly differ from the sequences of the group PFK_A (Figure 1; Figure S1 in Supplementary Material**)**. *OsPFK03* and *OsPFK09* were closely related to *AtPFK2* (At5g47810), and form the PFK_B group (Figure 1). The protein sequences of the PFK_B group are shorter than the other PFK sequences (Table 1; Figure S1 in Supplementary Material). *AtPFK2* is specifically expressed in seeds, but scarcely expressed in other tissues, and the same expression pattern was determined for both rice genes (Figure S4 in Supplementary Material; Winter et al., 2007). Nevertheless, in our transcript analysis *OsPFK03* was expressed at considerable amounts, while *OsPFK09* transcripts were present only at very low levels in vegetative tissues (Figure 2). All members of this sub-clade are localized to the cytosol (Figure 4, Mustroph et al., 2007). It is proposed that PFK_B members are characterized by distinct enzymatic properties for specific tissues, such as seeds and embryos. But, expression of AtPFK2 in tobacco leaves did not induce enhanced PFK activity under our assay conditions (Mustroph et al., 2007). Future analyses of *Arabidopsis* and rice mutants with deficiency in AtPFK2 and OsPFK03/09 expression could shed light on this topic.

The PFK_C subgroup proteins are targeted to plastids and include the rice genes *OsPFK07*, *OsPFK08*, and *OsPFK10* as well as *AtPFK5* (At2g22480; Mustroph et al., 2007; Figure 4). Indeed, PFK isoforms of various plant species were found in chloroplasts and in the cytosol (Cawood et al., 1988; Knowles et al., 1990; Turner and Plaxton, 2003). While cytosolic PFKs catalyze a step in the normal cytosolic glycolysis for energy metabolism, plastidal glycolysis using plastidal PFK contributes to starch breakdown and generation of metabolites for biosynthetic processes in dark-adapted or non-photosynthetic plastids (Plaxton, 1996). We propose that plastidal PFKs are light-dependently inactivated to avoid breakdown of photosynthates. But, we found significantly lower PFK activity in 24 h dark-incubated leaves and stems of rice plants exposed to ambient air (50% activity in comparison to light-grown plants, Table 2). It is possible that the *in vitro* activity does not reflect the *in planta* activity which could be influenced by redox regulation and phosphorylation (Kachru and Anderson, 1975; Cséke et al., 1982; Heuer et al., 1982).

### PFK and PFP gene expression under anoxia

The function of the two different phosphofructokinases in plants is still a matter of debate. It was hypothesized that plants might use PFP instead of PFK for the phosphorylation of fructose-6-phosphate during ATP deficiency (Weiner et al., 1987; Mertens et al., 1990; Stitt, 1998). Our results show that anoxia-tolerant rice plants induce the expression of genes coding for both enzymes, PFK as well as PFP, during anoxia. *OsPFK04* is a *bona fide* inducible gene upon anoxia in all organs, whereas *OsPFK05* transcripts were moderately increased in stems and leaves (Figure 2). Induction of *OsPFK04* transcript occurred within 30 min of anoxic stress. This rapid induction resembles that of accumulating *PDC1* mRNA (Figure 2). It is suggested that rice has a sensitive and rapid signaling pathway for the detection of low-oxygen levels, most likely via post-translational and oxygen-dependent regulation of group VII ERF transcription factors (Gibbs et al., 2011; Licausi et al., 2011). However, such induction of *PFK* genes was also found for all other plant species observed (Figure 6), including low-oxygen-sensitive *Arabidopsis* (At4g26270, At4g32480) and soybean. Therefore, it can be proposed that induction of PFK is important for metabolism under oxygen deficiency in both, sensitive and tolerant plants.

Also *OsPFPA3* and *OsPFP-B* transcripts were clearly increased in rice under anoxia in light and darkness (Figure 2), mainly in stems and leaves, which are the tissues with the highest tolerance to anoxia (Mustroph et al., 2006a,b). As stems and leaves store more carbohydrates and ferment them during anoxic periods, their cells survive anoxia better than root cells. Furthermore, these tissues might be able to produce more adaptive proteins, including PFP against stress through enhanced availability of photosynthetic energy. Interestingly, while *PFPAs* encoding the regulatory subunit were not induced in low-oxygen sensitive *Arabidopsis* or soybean plants, the *OsPFPA3* was stronger induced in anoxic rice seedlings than the catalytic subunit *OsPFP-B* (Figures 2 and 5) indicating a high importance for modulation of enzyme activity under oxygen deficiency (Figure 5). These results favor the idea that PFP plays a role in the reorganization of metabolism during low-oxygen stress in anoxia-tolerant leaves, but not in sensitive *Arabidopsis* plants or rice roots. However, the particular role of PFP in plant metabolism remains open. This question should be addressed in transgenic rice plants displaying reduced PFP activity.

Transcripts of four *PFK* genes were found to be less abundant during anoxia compared to aeration (*OsPFK06, OsPFK07, OsPFK08, OsPFK10*; Figure 2). Remarkably, three of the four *PFK* genes are plastidic and belong to group PFK_C (Table 1). Also *Arabidopsis*, soybean and poplar PFK_C members were not induced under oxygen deficiency stress and hypothetically hint at reduced plastidal starch degradation and biosynthetic processes under oxygen deficiency. However, rice seeds are able to germinate under anoxia by making use of the amylase-degraded starch (Guglielminetti et al., 1995; Perata et al., 1997). It is not entirely excluded that either the degradation products of starch are translocated from plastids as hexoses, or plastidal PFK activity is mainly post-translationally stimulated despite the transcriptional reduction. Thus, more detailed studies are needed to elucidate the link between reduced *OsPFK* transcription of plastid-localized isoforms and the response and adaptation to anoxic stress.

Illumination during the anoxic period greatly enhances the plant survival rates due to photosynthesis-driven ATP production (Mustroph et al., 2006b). In our recent analysis, we did not find dramatic differences in the induction of *phosphofructokinase* genes (Figure 2) or enzyme activities (Table 2) in light versus dark anoxia. This suggests that phosphofructokinase activity has not a major role in the positive effect of illumination during oxygen deficiency stress. But, all *phosphofructokinase* genes with reduced expression during an anoxic stress period showed an even stronger decrease in darkness than in light, especially in stems and leaves (Figure 2) suggesting an energy-dependent decrease of transcription in anoxia. It is reasonable to speculate on the avoidance of unwanted transcription and translation to save valuable ATP. It is reported that the highly energy-consuming translation of house-keeping genes is especially tightly regulated under oxygen deficiency stress (Branco-Price et al., 2008; Mustroph et al., 2009). In consistency, ADH and PDC activities did not increase as much during anoxia in darkness as in light (Table 2; Mustroph et al., 2006a,b).

Complete analyses of the expression of *phosphofructokinase* gene family under oxygen deficiency are only now possible after the identification of the entire gene family in rice. Previously, the effect of oxygen deficiency on the content of a single *phosphofructokinas*e transcript was studied by RNA blot analysis in rice (Umeda and Uchimiya, 1994; Minhas and Grover, 1999). Based on our classification of the *phosphofructokinase* genes, both research groups monitored *OsPFP-B* and concluded that the transcript level is strongly increased upon oxygen deprivation. This is in agreement with our results showing a two- to threefold increase in the *OsPFP-B* mRNA content (Figure 2). Data from recent microarray analyses using rice coleoptiles exposed to anoxic stress (Lasanthi-Kudahettige et al., 2007; Narsai et al., 2009), and of rice leaves to submergence (Mustroph et al., 2010) confirmed the highest accumulation of *OsPFK04* and *OsPFK05* transcript levels among the *phosphofructokinase* genes (Figure 6; Table S3 in Supplementary Material). The microarray data also show a moderate induction of *OsPFPA3* and *OsPFP-B* (Figure 5), as shown in our experiments (Figure 2). Furthermore, the reduced accumulation of *OsPFK06, OsPFK08, OsPFK10*, and *OsPFPA1* transcripts during anoxia in comparison to the aerated controls was congruently presented in the microarray profiles and our studies. When expression patterns, for example from *OsPFK01* and *OsPFPA4*, differ between reported and our own studies, these differences likely refer to the use of different plant tissues and rice cultivars as well as different growth conditions and stress applications.

### Induced expression of phosphofructokinase genes does modify enzyme activities only slightly

Despite the fact that some *PFK* and *PFP* genes were strongly expressed during anoxia in rice compared to aeration, the activities of PFK and PFP in cell extracts were only slightly increased in response to anoxic treatment (Table 2). PFP activity increased slightly but not significantly after 24 h of anoxia in leaves, which correlated with increased *OsPFPA3* transcript levels, while PFK activity was slightly induced in stems during illuminated anoxia. Although the translational activity of single mRNAs and the stability of individual isoforms are not known, it is proposed that the simultaneous increase of transcript amounts for two *PFK* and two *PFP* genes and the decrease of transcript amounts for four *PFK* and three *PFP* genes contribute to the maintenance of the activity of both enzymes during anoxia (Figure 2). However, rice shoots, the most tolerant tissue in this study, showed an increase in PFP enzyme activity during long-term anoxia. This elevated activity was never obtained for the anoxia-sensitive *Arabidopsis* plants (data not shown) and could presumably partly contribute to anoxia tolerance.

Increased activities of PFP, but not of PFK were reported for rice coleoptiles (Mertens et al., 1990; Kato-Noguchi, 2002) or suspension cells (Mohanty et al., 1993) in response to oxygen deficiency. Two reasons could explain the lower activation of enzyme activity in the present experiments. First, plant organs respond differently to oxygen deficiency stress. Coleoptiles possess the highest anoxia tolerance among the different plant tissues and can survive several days of anoxia. Therefore, it is possible that among other factors this tolerance is due to strong increase of PFP activity. Second, the experimental conditions differ in the studies. Rice suspension cells were exposed to 12 and 24 h of anoxia in darkness with carbohydrate addition, and a sixfold PFP activity increase was determined, but no increase of PFK activity (Mohanty et al., 1993). Without additional sugar supply, dark-incubated rice seedlings do not tolerate anoxia for more than 24 h before they die (Mustroph et al., 2006a). It is likely that the sugar supplement enables the strong increase in enzyme activity in the previous study (Mohanty et al., 1993).

We conclude that, although the overall *in vitro* activity of PFK and PFP was only slightly modified during anoxic treatment, the induction and repression of several of the *PFK* and *PFP* genes contributes to changes of the *in planta* metabolic activity. The encoded phosphofructokinases could have different affinities to substrates and cofactors, such as fructose-6-phosphate, ATP, and PPi. Furthermore, PFK and PFP activities are highly regulated by other metabolites like magnesium or phospho*enol*pyruvate (summarized in Plaxton, 1996). Another possible factor is phosphate, a potent activator of cytosolic PFK activity that functions as an inhibitor of plastidic PFK activity (Kelly and Latzko, 1977). In conclusion, the distinct upregulation of *PFP* genes during anoxia in rice provides a means for the use of PPi instead of ATP for the conversion of fructose-6-P to fructose-1,6-BP. An increase in *PFP* transcript levels and PFP activity during anoxia in rice likely alleviates the energy crisis. These regulatory mechanisms were not observed in low-oxygen sensitive plants, such as *Arabidopsis*, soybean, or poplar.

## Conflict of Interest Statement

The authors declare that the research was conducted in the absence of any commercial or financial relationships that could be construed as a potential conflict of interest.

## Supplementary Material

The Supplementary Material for this article can be found online at http://www.frontiersin.org/Plant_Physiology/10.3389/fpls.2013.00125/abstract

Supplementary Figure S1 **Alignment of amino acid sequences of PFKs from Arabidopsis and rice**. The sequences were aligned by use of the ClustalW method (http://www.genome.jp/tools-bin/clustalw). Similar amino acids are marked with shadows. The letters A, B, and C mark the three PFK subgroups. For the alignment, the corrected protein sequences for LOC_Os08g34050 (*OsPFK07*) and LOC_Os09g24910 (*OsPFK08*) were used (see Figure S2 in Supplementary Material).

Supplementary Figure S2 **Modified nucleotide and protein sequences for LOC_Os08g34050 (OsPFK07), LOC_Os09g24910 (OsPFK08), and LOC_Os09g12650 (OsPFPA4) after sequencing of several PCR products and comparison to the annotated sequences**. For *OsPFK07*, two versions were found in different varieties, one for Nipponbare (as well as Cigalon, M202, Dongjin, Hwayoung), and one for FR13A (as well as CT6241).

Supplementary Figure S3 **Subcellular localization of PFK isoforms from rice**. Full-length mRNA sequences or the N-terminal part of the sequences were cloned in frame in front of a GFP-coding sequence and were transiently transformed into tobacco leaves by Agrobacterium infiltration. After 3–4 days, protoplasts or undigested leaf disks were isolated and analyzed by confocal microscopy. Green color represents GFP fluorescence, red color represents chlorophyll autofluorescence. Full-length CDS: **(A)** OsPFK01; **(B)** OsPFK02; **(C)** OsPFK04; **(D)** OsPFK05; **(E)** OsPFK06; **(I,J)** OsPFK03; **(O)** OsPFK09; **(S)** OsPFK08; **(T)** OsPFK10. N-terminal part: **(F–H)** OsPFK03; **(K–N)** OsPFK09; **(P–R)** OsPFK05.

Supplementary Figure S4 **Expression of the PFK_B group members in different organs**. eFP browser pictures were obtained through http://bar.utoronto.ca (Winter et al., 2007). Red color intensity shows high expression level in the respective tissue type.

Supplementary Table S1 **Primers used for the experiments**. Actin primers were those used by Ren et al. (2005).

Supplementary Table S2 **Semi-quantitative RT-PCR analysis of rice phosphofructokinase genes: conditions for the PCR reactions**.

Supplementary Table S3 **Data for differential gene expression of phosphofructokinase genes in rice under oxygen-deficient conditions, obtained by microarray analyses with the Affymetrix rice Microarray Chip (Lasanthi-Kudahettige et al., 2007; Narsai et al., 2009; Mustroph et al., 2010)**. The signal-log-ratio (SLR) of all probe-sets is shown for each sample set. Data had been analyzed previously by GC-RMA.

## Footnotes

^1^http://rice.plantbiology.msu.edu

^2^http://rice.plantbiology.msu.edu

^3^http://rice.plantbiology.msu.edu

^4^http://www.phytozome.net/

## Abbreviations

ADH, alcohol dehydrogenase; PDC, pyruvate decarboxylase; PFK, ATP-dependent phosphofructokinase; PF2K, phosphofructo-2-kinase; PFP, pyrophosphate-fructose-6-phosphate-phosphotransferase; PPi, pyrophosphate.
