# Supplementary material for: Characterization of the Phosphofructokinase Gene Family in Rice and Its Expression Under Oxygen Deficiency Stress
Source: Front Plant Sci. 2013 May 14;4:125. doi: 10.3389/fpls.2013.00125 (PMC3653104; doi:10.3389/fpls.2013.00125)
Supplement: Supplementary Figure S1 — Alignment of amino acid sequences of PFKs from Arabidopsis and rice. The sequences were aligned by use of the ClustalW method (http://www.genome.jp/tools-bin/clustalw). Similar amino acids are marked with shadows. The letters A, B, and C mark the three PFK subgroups. For the alignment, the corrected protein sequences for LOC_Os08g34050 (OsPFK07) and LOC_Os09g24910 (OsPFK08) were used (see Figure S2 in Supplementary Material). [file 47628_Mustroph_DataSheet1.DOC]

**Supplemental Table 1:** Primers used for the experiments. Actin primers were those used by Ren *et al.* (2005).

| **Locus** | **Primer forward (5’🡪 3’)** | **Primer reverse (5’🡪 3’)** |
| --- | --- | --- |
| **RT-PCR** |  |  |
| Actin | TCCATCTTGGCATCTCTCAG | GTACCCTCATCAGGCATCTG |
| PDC1, Os05g39310 | CATCGGCTGCTGCAATGAGC | AGCCCACGCTGATGTAGACG |
| PFK01, Os01g09570 | TACCCAGAAAGGTGCTTCCGTA | CATCTTGAGTATCCACAAAATTC |
| PFK02, Os01g53680 | CAGAAGGGAGCATATGAAATATAT | GCATCTATTTGATCTGCTGC |
| PFK03, Os04g39420 | AACAATGAGAGGAGCTGTGGC | GTCCTGCTCCCGCTTTTGATCG |
| PFK04, Os05g10650 | ACGATCCACGCCGAGGTGCA | GTCGTGCGCGTCGGCGAGG |
| PFK05, Os05g44922 | AGAAGGGTGCATATGAGATCTTC | CATCTTGCTGCTCAGAAGTAGAT |
| PFK06, Os06g05860 | TACTCAAAGGGGTGCAGGAGTG | ATCTTTCCCCATTGATTCCTTTG |
| PFK07, Os08g34050 | CGTGCTTGGAGGAAATGGTAC | ACACAGAATCCTTTGGTCTTTAA |
| PFK08, Os09g24910 | TGTAATTGGTGGAAACGGTAG | ACACAAACCACGCAAAATCCC |
| PFK09, Os09g30240 | GTTCAACCAGGTGTACGCCG | GTGGTTCTTGGGATGAGACG |
| PFK09b, Os09g30240 | GCCTCTTCGACTTCCTCTAC | CGAGGTAGACAACAAACGAC |
| PFK10, Os10g26570 | TCATGCTGGAGCTAACCTTATA | TGCATCAGTTGCGTTTGACTG |
| PFPA1, Os02g48360 | CCGGAAGACGGCTAATGTCCC | CCAACTTCAAAGCCTGACAACTAGC |
| PFPA2, Os06g22060 | AGGAAGACTGCTGCTGTGCCG | AACTTCAAGGCTTGGCAAGCAGT |
| PFPA3, Os08g25720 | TTGTCAGCTTCGTCGCGCC | CCAAGTTGAGGTTGTTGCATGTAGT |
| PFPA4, Os09g12650 | TTCCTGAAGCCGAACAAGATG | CCAAGTCAAGATCACAGCATGTTGA |
| PFPB, Os06g13810 | ATGAAAACTCGTGTCATTGG | GTTTCAATTTTTGCAACCTG |
|  |  |  |
| **Cloning** |  |  |
| OsPFK01_GW | AAAAAGCAGGCTATGGAGGCCGTCGGCGTGGC | AGAAAGCTGGGTCACTGGCGCTGCTGTCCCGTTGC |
| OsPFK02_GW | AAAAAGCAGGCTATGGCGTCTCACATCATTCTG | AGAAAGCTGGGTTTTTCTCACCATTTGCCGGCTTC |
| OsPFK03_GW | AAAAAGCAGGCTATGAGAGGAGCTGTGGCCAT | AGAAAGCTGGGTTGTATTTTGGCTTCATGAAATCTGG |
| OsPFK04_GW | AAAAAGCAGGCTATGGAGGCCGCCACGGTTGTC | AGAAAGCTGGGTTGGCGGCGCCGTTGCAGAGG |
| OsPFK05_GW | AAAAAGCAGGCTATGGCGTCCTCCCACATCATC | AGAAAGCTGGGTTTATCTGGCTGTTCGACGAAGATGA |
| OsPFK06_GW | AAAAAGCAGGCTATGGCGTCGCCTCCAACCGC | AGAAAGCTGGGTTCTTCACAGCCTTGCCATTGGAAGC |
| OsPFK07_GW | AAAAAGCAGGCTATGGCTGTTTCTTTAAAATCA | AGAAAGCTGGGTTATGGAAATCAGGCTGGCCAGT |
| OsPFK08_GW | AAAAAGCAGGCTATGACCTTTTCTGGGATGGAC | AGAAAGCTGGGTTGTGGAAGTCCGGTTGGCCAGTG |
| OsPFK09_GW | AAAAAGCAGGCTATGGACGACGACGGCGAGACG | AGAAAGCTGGGTTGCTCGTCGGACCGGCCCGGATA |
| OsPFK10_GW | AAAAAGCAGGCTATGGCTCTAAAATCACCAGTG | AGAAAGCTGGGTTGTGGAAGTCTGGTTGCCCTGT |
| OsPFK07_2_GW | AAAAAGCAGGCTATGGCTGTTTCTTTAAAATCA | AGAAAGCTGGGTAGTATCACATTTCCAGATGCATC |
| PFK01_GW_short | AAAAAGCAGGCTATGGAGGCCGTCGGCGTGGC | AGAAAGCTGGGTTGAAATGGGTGCCCCTAGCAC |
| PFK02_GW_short | AAAAAGCAGGCTATGGCGTCTCACATCATTCTG | AGAAAGCTGGGTCGGAGAGGTAGTCCGTGAAG |
| PFK03_GW_short | AAAAAGCAGGCTATGAGAGGAGCTGTGGCCAT | AGAAAGCTGGGTCCCCCTTTCCTTCAAGATAGA |
| PFK04_GW_short | AAAAAGCAGGCTATGGAGGCCGCCACGGTTGTC | AGAAAGCTGGGTTGTGCACCACGATCTTCTTC |
| PFK05_GW_short | AAAAAGCAGGCTATGGCGTCCTCCCACATCATC | AGAAAGCTGGGTAGGCTGGATGATCTTGTAGTG |
| PFK06_GW_short | AAAAAGCAGGCTATGGCGTCGCCTCCAACCGC | AGAAAGCTGGGTATTGCTTCACAACCGAGTATGC |
| PFK07_GW_short | AAAAAGCAGGCTATGGCTGTTTCTTTAAAATCA | AGAAAGCTGGGTGTGCTCGATCATCGCTGTTCA |
| PFK08_GW_short | AAAAAGCAGGCTATGACCTTTTCTGGGATGGAC | AGAAAGCTGGGTACAGAAGTGCACGGTCATCCTT |
| PFK09_GW_short | AAAAAGCAGGCTATGGACGACGACGGCGAGACG | AGAAAGCTGGGTGCTCTCGCACGACGGTGTT |
| PFK10_GW_short | AAAAAGCAGGCTATGGCTCTAAAATCACCAGTG | AGAAAGCTGGGTCTCCAGCAGATGTTGGTGAAG |
|  |  |  |
| **sequencing** |  |  |
| OsPFK07_seq | GGGCAGGTTGATGTCTGTTTA | ATCAGCAGGAACACCAATGTC |
| OsPFPA1_seq | CTAATGTCCCCGATGCTACTG | ATGTCATGATCGCCAATGTCT |
| OsPFPA2_seq | CTCAGGAAGACTGCTGCTGTG | TTGTTTGATCCTCCCCATGTA |
| OsPFPA3_seq | CAACGGTGAAGGTGGAGTATG | AATGTCGCCCATGTAGTCTTG |
| OsPFPA4_seq | AGAGGAGGGCTCTGTATCAGC | CCGACATAACCTTCAGCACAT |
|  |  |  |

**Supplemental Table 2:** Semi-quantitative RT-PCR analysis of rice *phosphofructokinas*e genes: conditions for the PCR reactions.

| Locus | Name | Length of fragment | Temperature for PCR | Cycle number | Special remarks and additives |
| --- | --- | --- | --- | --- | --- |
|  |  |  |  |  |  |
|  | actin | 337 | 54 | 28 |  |
| Os05g39310 | PDC1 | 371 | 54 | 28 | 1 M Betaine |
| Os01g09570 | OsPFK01 | 440 | 54 | 35 |  |
| Os01g53680 | OsPFK02 | 437 | 54 | 31 |  |
| Os04g39420 | OsPFK03 | 439 | 54 | 30 |  |
| Os05g10650 | OsPFK04 | 420 | 66 | 41 | half MgCl_2_ concentration |
| Os05g44920 | OsPFK05 | 327 | 54 | 28 |  |
| Os06g05860 | OsPFK06 | 436 | 54 | 28 |  |
| Os08g34050 | OsPFK07 | 270 | 54 | 34 |  |
| Os09g24910 | OsPFK08 | 396 | 54 | 34 |  |
| Os09g30240 | OsPFK09 | 453 | 54 | 41 |  |
| Os10g26570 | OsPFK10 | 427 | 54 | 36 | 0.5% DMSO |
| Os02g48360 | OsPFPA1 | 338 | 54 | 36 | 0.5% DMSO |
| Os06g22060 | OsPFPA2 | 335 | 54 | 32 | 0.5% DMSO |
| Os08g25720 | OsPFPA3 | 339 | 54 | 40 | 0.5% DMSO |
| Os09g12650 | OsPFPA4 | 337 | 54 | 40 | 0.5% DMSO |
| Os06g13810 | OsPFP_B | 569 | 54 | 41 |  |

**Supplemental Table 3:** Data for differential gene expression of phosphofructokinase genes in rice under oxygen-deficient conditions, obtained by microarray analyses with the Affymetrix rice Microarray Chip (Lasanthi-Kudahettige et al., 2007, Narsai et al., 2009, Mustroph et al., 2010). The signal-log-ratio (SLR) of all probe-sets is shown for each sample set. Data had been analyzed previously by GC-RMA.

See Excel file

**Supplemental Figure 1:** Alignment of amino acid sequences of PFKs from Arabidopsis and rice. The sequences were aligned by use of the ClustalW method (http://www.genome.jp/tools-bin/clustalw). Similar amino acids are marked with shadows. The letters A, B and C mark the three PFK subgroups. For the alignment, the corrected protein sequences for LOC_Os08g34050 (*OsPFK07*) and LOC_Os09g24910 (*OsPFK08*) were used (see **Supplemental Figure 2**).

10 20 30 40 50 60 70

....|....|....|....|....|....|....|....|....|....|....|....|....|....|

A

B

C

**LOC_Os01g09570.1**  ---------------------------------------MEAVGVAPAPAGVPEKKLLEVKESRKAAPAA

**LOC_Os05g10650.1**  --------------------------------MEAATVVAAPIPAADAAAKALEKKLLDLELPPFPAPAK

**AT4G32840.1**  ----------------------------------------------------------------------

**AT4G26270.1**  ----------------------------------------------------------------------

**AT5G56630.1**  ----------------------------------------------------------------------

**LOC_Os06g05860.1**  -------MASPPTASASASEAAESGRRSAPGPIDVPSPRDHLHHLLDRRDTPRVVHVEGTTMQRQRGEAA

**AT4G29220.1**  ----------------------------------------------------------------------

**LOC_Os01g53680.1**  -MASHIILPKEEEAALGVAVEEDHDSPAAPG---YQHQQGPPVAKALPFSATCVRISRDSYPNLRALRNA

**LOC_Os05g44922.1**  MASSHIILPPDDDDEEQRRLEEEEDEDPWARSGPPPAAPEHAMKAALPFSATCVRISRDSYPNLRALRNA

**AT5G61580.1**  -----------------------MEASISFLGSTKPNISLFNPSSNVLPRRDFPLPALKLKKVSVLPRIL

**AT5G47810.1**  ----------------------------------------------------------------------

**LOC_Os04g39420.1**  ----------------------------------------------------------------------

**LOC_Os09g30240.1**  ----------------------------------------------------------------------

**LOC_Os08g34050.2**  ------------MAVSLKSSGSFCSTPPQWLHSTRDRILYGYSHSNAKECTCKKTKRPAPLCVKATST-K

**LOC_Os09g24910.2**  -------MTFSGMDIALKASTHSSTSQQHWLHSTRYRCQYGLGST----HLNGRKRSPMVLSVRAVSG-K

**LOC_Os10g26570.1**  -----------------MALKSPVDFAGSITSGQKDPCCFGVPGCNPRCVRYNKKSRTCRLVTRAISVDR

**AT2G22480.1**  -------MDALSQAISSGISVPYKNNSSSLVPSHGLTSLILRKSRSPVNPSSRSRVSVRASEIQHSKTSA

**Clustal Consensus**

80 90 100 110 120 130 140

....|....|....|....|....|....|....|....|....|....|....|....|....|....|

**LOC_Os01g09570.1**  PSTSMAAKWAMKKKLVGGDAGYVLEDVPHLTDYLPELPTYPNPLQDNPAYSVVK--------QYFVNTDD

**LOC_Os05g10650.1**  KAAAKVVAAAPKKKLAGGAGGYVLEDVPHLTDYLPNLPSFPNPLQNHPAYSVVK--------QYFVNADD

**AT4G32840.1**  ---MASNGVDEQIKLVEGPAGYVLEDVPHLSDYILDLPTYPNPLQSNAAYSVVR--------QYFVDEDD

**AT4G26270.1**  ----MSTVESSKPKIINGSCGYVLEDVPHLSDYLPGLPTYPNPLQDNPAYSVVK--------QYFVDADD

**AT5G56630.1**  ----MSSPRSNKPKIVNGPGGYILQDVPHLIDYLPDLPTYPNPLQDNPAYSVVK--------QYFVHADD

**LOC_Os06g05860.1**  GDAGAAAAAKPEVKLVTGDGGYVLEDVPHVCDYLPDLPTYSNPLQDNPAYSVCR--------QYFVNPDD

**AT4G29220.1**  ---MSSSVPNSDRKIVTGPAGYILEDVPHFSDDFPDHPTYPNPLQDNAAYSVVK--------QYFVDEDD

**LOC_Os01g53680.1**  SAMSLPDDDAAYAKLEEGDYGYLLDDVPHFTDYLSDLPTFPNPLQDHPAYSTVK--------QYFVNADD

**LOC_Os05g44922.1**  SSVSLAD--AAYVKISEGDFGYVLDDVPHLVDHLPDAPTYPNPLQDHPAYSTVK--------QYFVNEDD

**AT5G61580.1**  HQKRLIRAQCSDGFKPEEDDGFVLEDVPHLTKFLPDLPSYPNPLKESQAYAIVK--------RTFVSSED

**AT5G47810.1**  ---------------------MAAETSIRKLPSLSGLRHRRNPLEDNPYFHPSN--------GFYITPSD

**LOC_Os04g39420.1**  ----------------------------------------------------------------------

**LOC_Os09g30240.1**  --------------MDDDGETPMPSLELHKLPTLAG-AAIPNPIARHPLYHPSP--------SFFISPTD

**LOC_Os08g34050.2**  VELDFNDPSWKQKFQEDWDKRFNLPRITDIYDLKPRPTTFSLKKNRSPAGDENG--TPMDKWNGYVNSDD

**LOC_Os09g24910.2**  SDLDFSDPSWKEKYQEDWNRRFSLPHITDIYDLKPRLTTFSLKKNRTDGGS-----LSADKWNGYVNKDD

**LOC_Os10g26570.1**  PQLDFSNSDWKKQFQEDFDRRFSLPHLKDVIDVEPRPTTFSLKS-RTPLENVNG--SMQGSWNGYVNDDD

**AT2G22480.1**  SSIDLSDPDWKLKYEKDFEQRFSIPHITDVLPDAEAIRSTFCLKMRSPTEDFVGGYPSDEEWHGYINNND

**Clustal Consensus**

150 160 170 180 190 200 210

....|....|....|....|....|....|....|....|....|....|....|....|....|....|

**LOC_Os01g09570.1**  TVTQKIVVHKT-------------SARGTHFRRAGPRQRVYFQSDEVNAAIVTCGGLCPGLNTVIRELVC

**LOC_Os05g10650.1**  TVAKKIVVHKG-------------SARGTHFRRAGPRQRVFFQPDEVSAAIVTCGGLCPGLNTVIRELVC

**AT4G32840.1**  TVQEKIVVHKD-------------SPRGTHFRRAGPRQKVYFKPSDVRACIVTCGGLCPGLNTVIREIVC

**AT4G26270.1**  SVPQKIVVHKD-------------GPRGIHFRRAGPRQKVYFESDEVHACIVTCGGLCPGLNTVIREIVS

**AT5G56630.1**  SVPEKVVVHKD-------------GPRGVHFRRAGPRQKVYFESDEVHACIVTCGGLCPGLNTVIREVVS

**LOC_Os06g05860.1**  TVCQKAIVHKD-------------GPRGNHFRRAGPRQRVFFESDEVHACIVTCGGLCPGLNTVIREIVC

**AT4G29220.1**  TVPQKIVVHPD-------------SPRGTHFRRAGPRQRVYFESDDVLACIVTCGGLCPGLNTVIREIVC

**LOC_Os01g53680.1**  TVPEKVVVQKD-------------SPRGVHFRRAGPRQRVYFESEDVKACIVTCGGLCPGLNTVIRELVC

**LOC_Os05g44922.1**  TVPQKVVVQKN-------------SRRGVHFRRAGPRQKVYFESDEVKACIVTCGGLCPGLNTVIRELVC

**AT5G61580.1**  VVAQNIVVQKG-------------SKRGVHFRRAGPRERVYFRSDEVKACIVTCGGLCPGINTVIREIVC

**AT5G47810.1**  VILAQVAYDHS-----------AHSQSRVAYHRAGPRREIMYEPSAVKAAIVTCGGLCPGMNTVIRELVV

**LOC_Os04g39420.1**  ----------------------------------------------------------------------

**LOC_Os09g30240.1**  VVLRDILFDASPASA----AGERRRRHVAAYHRAGPRREVAFDPATVRAAIFTCGGLCPGTNTVVRELVV

**LOC_Os08g34050.2**  RALLKVIKYSSPNSAGAECIDPDCSWVEQWVHRAGPRKEIYYEPEEVKAAIVTCGGLCPGLNDVIRQIVF

**LOC_Os09g24910.2**  RALLKVIKYASPTSAGAECVDPDCSWVEHWIHRAGPRKEIYYEPAEVKAAIVTCGGLCPGLNDVIRQIVF

**LOC_Os10g26570.1**  RALLKVIKFASPTSAGADCIDPDCSWVEQWVHRAGPRKQIYFEPQYVKAGIVTCGGLCPGLNDVIRQIVL

**AT2G22480.1**  RVLLKVISYSSPTSAGAECLDHDCSWVEQWIHRAGPREKIYFRPEEVKAAIITCGGLCPGLNDVIRHIVI

**Clustal Consensus**

220 230 240 250 260 270 280

....|....|....|....|....|....|....|....|....|....|....|....|....|....|

**LOC_Os01g09570.1**  GLYDMYGVT-SVVGIEGGYKGFYSR--NTVALTPKSVNDIHKRGGTVLGTSRGGHDTGKIVDSIKDRGIN

**LOC_Os05g10650.1**  GLHDMYGVT-SVVGIEGGYRGFYAR--NTVELTPRSVNGIHKRGGTVLGTSRGGQDTGKIVDSIQDRGIN

**AT4G32840.1**  GLHFMYGVT-EVIGVDCGFRGFYSK--NTVALTPKTVSDIHKRGGTILGTSRGGHDTSKIVDNIQDREIN

**AT4G26270.1**  SLSYMYGVK-RILGIDGGYRGFYAK--NTVSLDSKVVNDIHKRGGTILGTSRGGHDTTKIVDSIQDRGIN

**AT5G56630.1**  SLSYMYGVK-RILGIDGGYRGFYAK--NTIPLNSKVVNDIHKRGGTIIGTSRGGHDTNKIVDSIQDRGIN

**LOC_Os06g05860.1**  GLYDMYGVS-RVLGIQGGYRGFYAC--NTIDLSPKSVNDIHKRGGTVLGTSRGGHDTMKIVDSIQDRGIN

**AT4G29220.1**  GLSYMYGVK-RILGIDGGYRGFYAR--NTIHLDLKTVNDIHRSGGTILGTSRGGHNTTKIVDSIQDRGIN

**LOC_Os01g53680.1**  GLSHMYNVN-DIFGIQNGYKGFYSS--NYLPMTPKSVNDIHKRGGTVLGTSRGGHDTKKIVDNIQDRGIN

**LOC_Os05g44922.1**  GLAHMYNVS-KIYGIQNGYKGFYSS--NYLTLTPKSVDDIHKRGGTVLGTSRGGHDTKKIVDNIQDRGIN

**AT5G61580.1**  GLNNMYGVN-NILGIQGGYRGFYSK--NTMNLTPKVVNDIHKRGGTFLQTSRGGHDTAKIVDNIQDRGIN

**AT5G47810.1**  GLWELYGVR-EIYGIPAGYRGFYSM--KAVKLDPKAVHDWHKKGGTVLATSRGGFHLQKIVDAIHLNGYN

**LOC_Os04g39420.1**  ----------------------------------------------------------------------

**LOC_Os09g30240.1**  GLSELYGVRGGVFGVRNGYRGFYSD--EVVPLDPAAVEHWHKAGGAALGTSRGGFDLARIVDAIERHGFN

**LOC_Os08g34050.2**  TLETYGVKN--IVGIPFGYRGFFEKGLKEMPLSRHLVENINLAGGSFLGVSRGGAKTSEIVDSIQARRID

**LOC_Os09g24910.2**  TLEIYGVKN--IVGIQFGYRGFFEKGLKEMPLSRKVVENINLSGGSFLGVSRGGAKTSEIVDSIQARRID

**LOC_Os10g26570.1**  TLEKYGVKN--IVGIQHGFRGFFEDHLAEVPLNRQVVQNINLAGGSFLGVSRGGANISDIVDSIQARRLD

**AT2G22480.1**  TLEIYGVKN--IVGIPFGYRGFSDKDLTEMPLSRKVVQNIHLSGGSLLGVSRGGPSVSEIVDSMEERGIN

**Clustal Consensus**

290 300 310 320 330 340 350

....|....|....|....|....|....|....|....|....|....|....|....|....|....|

**LOC_Os01g09570.1**  QVYIIGGDGTQKGASVIYEEVRRRG-LKCSVVGVPKTIDNDIAVIDKSFGFDTAVEEAQRAINAAHVEAE

**LOC_Os05g10650.1**  QVYIIGGDGTQKGAATIHAEVQRRG-LKCAVVGVPKTIDNDIAVIDRSFGFDTAVEEAQRAINAAHVEAE

**AT4G32840.1**  QVYIIGGDGTQKGANAIYKEIRRRG-LKVAVAGIPKTIDNDIPVIDKSFGFDTAVEEAQRAINAAHVEAT

**AT4G26270.1**  QVYIIGGDGTQRGASVIFEEIRRRG-LKVAVIGIPKTIDNDIPVIDKSFGFDTAVEEAQRAINAAHVEAE

**AT5G56630.1**  QVYIIGGDGTQRGASVIFEEIRRRR-LKVAVVGIPKTIDNDIPVIDKSFGFDTAVEEAQRAINAAHVEAE

**LOC_Os06g05860.1**  QVYVIGGDGTQRGAGVIFEEIRRRG-LKVAVAGIPKTIDNDIPVIDRSFGFDTAVEEAQRAINAAHVEAG

**AT4G29220.1**  QVYIIGGDGSQKGAAAIFEEIRKRK-LKVAVAGIPKTIDNDIPIIDRSFGFDTAVEEAQRAINAAHVEAT

**LOC_Os01g53680.1**  QVYIIGGDGTQKGAYEIYKEIRRRG-LKVAVAGVPKTIDNDIAVIDKSFGFDSAVEEAQRAIDAAHVEAS

**LOC_Os05g44922.1**  QVYIIGGDGTQKGAYEIFKEIRKRG-LKVSVAGIPKTIDNDIAIIDKSFGFDTAVEEAQRAIDSAHVEAC

**AT5G61580.1**  QVYIIGGGGTQKGAEKIYEEVERRG-LQVAVSGIPKTIDNDIAVIDKSFGFDTAVEEAQRAINAAHVEVE

**AT5G47810.1**  QVYIIGGDGTMRGAVEIFKEISLRK-LEVGITVIPKTVDNDVGIIDRSFGFQTAVEMAQEAISAAHVEAE

**LOC_Os04g39420.1**  ----------MRGAVAIFNEFKRRG-LNISITGIPKTVDNDIGIIDRSFGFQTAVEIAQQAIDAAHVEAV

**LOC_Os09g30240.1**  QVYAVGGDGTMRGAARIHREVRRRGRLAVAVAGIPKTVDNDVGVVDRSFGFHTAVEAAQQAIAAGHVEAE

**LOC_Os08g34050.2**  MLFVLGGNGTHAGANAIHEECRKRK-LKVSVVAVPKTIDNDILLMDKTFGFDTAVEEAQRAINSAYIEAR

**LOC_Os09g24910.2**  MLFVIGGNGSHAGANAIHEECRKRK-LKVSVVAVPKTIDNDILFMDKTFGFDTAVEEAQRAINSAYIEAR

**LOC_Os10g26570.1**  MLFVLGGNGTHAGANLIHEECRKRK-LKVSIVGVPKTIDNDILLMDKTFGFDTAVEAAQRAINSAYIEAH

**AT2G22480.1**  MLFVLGGNGTHAGANAIHNECRKRK-IKVAVVGVPKTIDNDILHMDKTFGFDTAVEEAQRAINSAYIEAH

**Clustal Consensus** ** *. * * : .: :***:***: :*::***.:*** **.** :.::*.

360 370 380 390 400 410 420

....|....|....|....|....|....|....|....|....|....|....|....|....|....|

**LOC_Os01g09570.1**  SAENGIGVVKLMGRNSGFIAMYATLASRDVDCCLIPESPFYLEGKGGLLEFIEKRLKDNGHMVIVVAEGA

**LOC_Os05g10650.1**  SAENGVGVVKLMGRNSGFIAMYATLASRDVDLCLIPESPFYLEGKGGLLEFAEKRLRENGHMVIVVAEGA

**AT4G32840.1**  SVENGIGIVKLMGRYSGFIAMYATLASRDVDCCLIPESPFYLEGKGGLYEFIAKRLRENGHMVIVIAEGA

**AT4G26270.1**  SIENGIGVVKLMGRYSGFIAMYATLASRDVDCCLIPESPFYLEGEGGLFEYIEKRLKESGHMVLVIAEGA

**AT5G56630.1**  SNENGIGFVKLMGRYSGYIAMYATLASRDVDCCLIPESPFYLEGEGGLFEFIERRLKDHGHMVIVLAEGA

**LOC_Os06g05860.1**  SAENGIGLVKLMGRHSGFIAHYATLASRDVDCCLIPESPFYLEGEGGLFRYLEKRLKENGHMVIVVAEGA

**AT4G29220.1**  SFENGIGLVKLMGRYSGFIAMHATLASRDVDCCLIPESPFFLEGSGGLFEFIDKRLKESGHMVIVIAEGA

**LOC_Os01g53680.1**  SAENGIGLVKLMGRYSGFIAMYATLASRDVDCCLIPESPFYLEGEGGLFEYIEKRLKENNHMVIVVAEGA

**LOC_Os05g44922.1**  SAENGIGLVKLMGRYSGFIAMYATLASRDVDCCLIPESPFYMDGEGGLLQYVERRLKENKHMVIVVAEGA

**AT5G61580.1**  SVENGVGIVKLMGRYSGFIAMIATLANRDVDCCLIPESPFFLEGKGGLFEFIEERLKENRHMVIVIAEGA

**AT5G47810.1**  SAVNGIGLVKLMGRSTGHIALHATLSSRDVDCCLIPEMDFYLEGKGGLFEFLEKRLKERGHAVLVVAEGA

**LOC_Os04g39420.1**  SAVNGIGLVKLMGRSTGHIALHATLSSRDVDCCLIPEVDFYLEGKGGLFEFLYERIKQKGHAVVVVAEGA

**LOC_Os09g30240.1**  SAANGVGLVKLMGRSAGHIALHATLSSRDVDCCLIPEEDFYLRGAGGLFDFLYRRIKDNGHAVVVVAEGA

**LOC_Os08g34050.2**  SAYHGIGLVKLMGRSSGFIAMHASLSSGQVDVCLIPEVPFTLDGEYGVLRHLEHLLKTKGFCVVCVAEAA

**LOC_Os09g24910.2**  SAYHGIGLVKLMGRSSGFIAMQASLSSGQIDVCLIPEVSFTLDGEHGVMRHLEHLLEKKGFCVVCVAEGA

**LOC_Os10g26570.1**  SAFHGIGLVKLMGRSSGFITMHASLSSGQVDICLIPEVPFTLDGPNGVLQHLEHLIETKGFALICVAEGA

**AT2G22480.1**  SAYHGIGVVKLMGRNSGFIAMQASLASGQVDICLIPEVPFNLHGPNGVLKHLKYLIETKGSAVICVAEGA

**Clustal Consensus** * :*:*.****** :*.*: *:*:. ::* ***** * : * *: . :. :: :**.*

430 440 450 460 470 480 490

....|....|....|....|....|....|....|....|....|....|....|....|....|....|

**LOC_Os01g09570.1**  GQDLIAKSMNFVDTQ----DAS-GNKLLLDVGLWLSQKIKDHFKKKR-NFPITLKYIDPTYMIRAVRSNA

**LOC_Os05g10650.1**  GQDVIARSMRLADAH----DAS-GNKVLLDVGLWLCAKIKDHFKKKA-NFPITLKYIDPTYMIRAVPSNA

**AT4G32840.1**  GQDLVAESIEQQ-------DAS-GNKLLKDVGLWMSLKIKEYFAKHN-VMDITLKYIDPTYMIRAIPANA

**AT4G26270.1**  GQDLMSKSMES-MTL----KDASGNKLLKDVGLWLSQSIKDHFNQK--KMVMNLKYIDPTYMIRAVPSNA

**AT5G56630.1**  GQDLMCKSMES-TPM----DAS-GNKLLKDVGLWLSQSIKDHFKKN--KMVMNLKYIDPTYMIRAVPSNA

**LOC_Os06g05860.1**  GQKLINETKES-MGK----DAS-GNSILLDVGLWLSQKIKEHFKKI--KTTINLKYIDPTYMIRAIPSNA

**AT4G29220.1**  GQDLLSESMKESTTL----KDASGNKLLQDIGLWISQRIKDHFAK---KMTLTLKYIDPTYMIRAVPSNA

**LOC_Os01g53680.1**  GQDLIAKSIAAADQI----DAS-GNKLLLDVGLWLTHKIKDYCKNK--KMEMTIKYIDPTYMIRAIPSNA

**LOC_Os05g44922.1**  GQDLIAKSLSTSEQQ----DAS-GNKLLLDIGLWLTHKIKDHFKSK--KMEMTIKYIDPTYMIRAIPSNA

**AT5G61580.1**  GQDYVAQSMRASETK----DAS-GNRLLLDVGLWLTQQIKDHFTNVR-KMMINMKYIDPTYMIRAIPSNA

**AT5G47810.1**  GQEMIPRNESQKQE-----RDESGNAVFLDVGVWFKSVLKAWWEREHPDELFTVKYIDPTYMIRAVPANA

**LOC_Os04g39420.1**  GQELIPRTDDQKRE-----QDESGNIVFLDVGPWLKSELGKWWKREHPSELFTVKYIDPTYMIRAVPANA

**LOC_Os09g30240.1**  GQRLIPRTTTTSASGACAGADESGNETFLDVGAWLKAEMRAWWEEEHAGEVFTVKYIDPTYMIRAVPANA

**LOC_Os08g34050.2**  GQELLQKSGATDASG---------NVILSDIGVHMQQKIKMHFKDIG--VPADVKYIDPTYMVRACRANA

**LOC_Os09g24910.2**  GQDLLQKSNATDASG---------NVILSDFGVHMQQKIKSHFKDIG--VPADVKYIDPTYMVRACRANA

**LOC_Os10g26570.1**  GQEHLQQSNATDASG---------NMILGDIGVHLHQKIKAHFKEIG--VHSDVKYIDPTYMVRAVRANA

**AT2G22480.1**  GQNFLEKTNAKDASG---------NAVLGDFGVYIQQETKKYFKEIS--TPIDVKYIDPTYMIRAVRANA

**Clustal Consensus** ** : .. * : *.* : :********:** :**

500 510 520 530 540 550 560

....|....|....|....|....|....|....|....|....|....|....|....|....|....|

**LOC_Os01g09570.1**  SDNVYCTLLAHSALHGAMAGYTGFTVAPVNGRHAYIPFYRITEKQNKVVITDRMWARVLCSTNQPCFLSH

**LOC_Os05g10650.1**  SDNVYCSLLAHSAIHGAMAGYTGFTVAPVNGRHAYIPFYRITEKQNKVVITDRMWARVLCSTNQPCFLST

**AT4G32840.1**  SDNVYSTLLAQSAVHGAMAGYTGFVSGLVNGRHTYIPFNRITERQNKVVITDRMWARMLSSTNQPSFMNP

**AT4G26270.1**  SDNVYCTLLAQSAVHGAMAGYTGYISGLVNGRQTYIPFYRITEKQNHVVITDRMWARLLSSTNQPSFLGP

**AT5G56630.1**  SDNVYCTLLAQSAVHGAMAGYTGYTSGLVNGRQTYIPFYRITETQNNVVITDRMWARLLSSTNQPSFLGP

**LOC_Os06g05860.1**  SDNVYCTLLAHSVVHGAMAGYTGFTVGQVNGRHCYIPFYRITEKQNKVSITDRMWARLLSSTNQPSFLSK

**AT4G29220.1**  SDNVCCTLLAQSAVHGVMAGYNGFTVGLVNGRHTYIPFNRITEKQNKVVITDRMWARLLSSTNQPSFMKQ

**LOC_Os01g53680.1**  SDNVYCTLLAHSAIHGAMAGYS-FTVGMVNGRHAYIPFHRVTSTRNKVKITDRMWARLLSSTNQPSFLSQ

**LOC_Os05g44922.1**  SDNVYCTLLAHSAIHGAMAGYS-FTVGNVNGRHAYIPFYRVTSTRNKVKITDRMWARLLSSTNQPSFLSQ

**AT5G61580.1**  SDNVYCTLLAQSAVHGAMAGYSGFTVGPVNSRHAYIPISQVTEVTNTVKLTDRMWARLLASTNQPSFLTG

**AT5G47810.1**  TDNLYCTLLAHSAIHGVMAGYTGFVPGPINGNYAYIPLEEVAQTKNQVNTRDHKWAWVRSVTNQPDFETN

**LOC_Os04g39420.1**  TDNLYCTLLAHSAIHGIMAGYTGFVPGPINGNYSYIPLEDVAVAKNPVDVNDHKWAWVRSVTNQPDFMKP

**LOC_Os09g30240.1**  GDNLYCTLLAHAAIHGAMAGYTGFVSGTINGNYAYIPMDEVAEAKNPVDTKDHKWAWVRSITNQPDFIRA

**LOC_Os08g34050.2**  SDAILCTVLGQNAVHGAFAGFSGITSCICNTHYVYLPITEVITVPKRVNPNSRMWHRCLTSTGQPDFH--

**LOC_Os09g24910.2**  SDAILCTVLGQNAVHGAFAGFSGITSGICNTHYAFLPITEVITKPKRVNPNSRMWHRCLTSTGQPDFH--

**LOC_Os10g26570.1**  SDAILCTVLGQNAVHGAFAGFSGITTGICNTHNVYLPISEVIKSTRFVDPNSRMWHRCLTSTGQPDFH--

**AT2G22480.1**  SDGILCTVLGQNAVHGAFAGYSGITVGIINTHYAYLPITEVIAYPKSVDPNSRMWHRCLTSTGQPDFI--

**Clustal Consensus** * : .::*.: .:** :**:. * . ::*: : . * .: * *.** *

570 580 590 600

....|....|....|....|....|....|....|....|....|

**LOC_Os01g09570.1**  EDVEHLKHDDDEHHLHNTQLLEGESSPVKDSSKCNGTAAPV----

**LOC_Os05g10650.1**  EDVEKAGQDDEEPIVPLVEGENSLVKAPPLLANAGDRAALCNGAA

**AT4G32840.1**  PKGTTEFTD------------------------------------

**AT4G26270.1**  KDVFD-NKEKPMSALLDDGNCNGVVDVPPVTKEITK---------

**AT5G56630.1**  KDTSEEKKELPETPLLDDG----AVDIPPVTKEVTK---------

**LOC_Os06g05860.1**  KDVEDAKMEEERASKFFDGPPPNPKVEDKVASNGKAVK-------

**AT4G29220.1**  ADKIHSNQLVGEPGTMKW---------------------------

**LOC_Os01g53680.1**  KDIDAAREADKLASKSPVPVNTKEHGENVKKPANGEK--------

**LOC_Os05g44922.1**  KDIDEAKENDRTANKPPLPTGLSHHVANSFDQSASSSSNSQI---

**AT5G61580.1**  EGALQNVIDMETQEKIDNMKISSI---------------------

**AT5G47810.1**  VKG------------------------------------------

**LOC_Os04g39420.1**  KY-------------------------------------------

**LOC_Os09g30240.1**  GPTS-----------------------------------------

**LOC_Os08g34050.2**  ---------------------------------------------

**LOC_Os09g24910.2**  ---------------------------------------------

**LOC_Os10g26570.1**  ---------------------------------------------

**AT2G22480.1**  ---------------------------------------------

**Clustal Consensus**

**Supplemental Figure 2:** Modified nucleotide and protein sequences for LOC_Os08g34050 (PFK07), LOC_Os09g24910 (PFK08) and LOC_Os09g12650 (PFPA4) after sequencing of several PCR products and comparison to the predicted sequences. For PFK07, two versions were found in different varieties, one for Nipponbare (as well as Cigalon, M202, Dongjin, Hwayoung), and one for FR13A (as well as CT6241).

>LOC_Os08g34050.2; OsPFK07_mod_Nipponbare

ATGGCTGTTTCTTTAAAATCAAGTGGCAGTTTTTGTAGCACACCGCCTCAGTGGCTGCATTCAACAAGGGATCGAATTTTATACGGTTATTCTCATTCAAATGCCAAAGAGTGCACTTGCAAGAAAACAAAAAGGCCTGCTCCACTGTGTGTTAAAGCTACTTCCACGAAAGTGGAATTAGATTTCAATGATCCATCTTGGAAGCAGAAGTTTCAGGAAGACTGGGATAAACGTTTTAATTTGCCACGTATTACAGACATATATGATTTGAAACCAAGGCCAACCACATTCTCACTCAAGAAAAACAGAAGTCCTGCAGGTGATGAAAATGGTACACCTATGGATAAATGGAATGGTTATGTGAACAGCGATGATCGAGCACTTTTGAAGGTGATAAAGTATTCCTCGCCTAACTCTGCTGGAGCAGAGTGCATTGATCCTGACTGTAGCTGGGTGGAACAATGGGTACATCGTGCAGGCCCTCGTAAGGAGATATACTATGAACCAGAGGAAGTAAAAGCTGCCATAGTTACTTGTGGAGGGCTCTGCCCTGGTTTGAACGATGTCATCAGACAGATAGTATTCACTCTAGAGACCTATGGGGTTAAGAATATTGTTGGGATTCCATTTGGTTATCGTGGATTTTTTGAAAAGGGCCTAAAAGAAATGCCACTTTCACGTCATCTGGTGGAGAACATAAATCTTGCTGGTGGAAGTTTTCTAGGAGTCTCTCGTGGAGGAGCTAAAACTAGTGAGATTGTAGATAGTATACAGGCCAGAAGAATTGATATGCTTTTCGTGCTTGGAGGAAATGGTACCCATGCAGGAGCAAATGCTATCCATGAAGAGTGCCGGAAGAGAAAGCTAAAAGTTTCAGTTGTAGCAGTTCCAAAGACCATCGACAATGATATACTTTTGATGGACAAAACATTTGGTTTCGATACGGCTGTTGAAGAAGCTCAGCGGGCCATTAATTCTGCATATATAGAGGCACGAAGCGCATACCATGGCATTGGTTTGGTCAAATTAATGGGAAGAAGCAGTGGCTTCATTGCAATGCATGCTTCCCTTTCAAGTGGGCAGGTTGATGTCTGTTTAATACCAGAGGTTCCTTTCACGCTTGATGGAGAATATGGTGTTCTACGACACCTTGAGCATTTGTTAAAGACCAAAGGATTCTGTGTTGTTTGTGTTGCTGAAGCTGCAGGACAAGAGTTTACAAAAATCAGGTGCAACAGATGCATCTGGAAATGTGATACTTAGTGACATCGGTGTTCATATGCAACAGAAGATTAAGATGCATTTCAAGGACATTGGTGTTCCTGCTGATGTAAAATACATTGATCCGACATATATGGTTCGGGCATGTCGTGCCAATGCATCTGATGCAATTTTGTGCACTGTACTTGGACAAAATGCTGTCCATGGAGCATTTGCTGGGTTCAGTGGCATCACTTCTTGCATCTGCAACACGCACTACGTCTACCTCCCCATCACAGAAGTCATAACAGTACCGAAGCGCGTGAACCCTAATAGCAGGATGTGGCACCGTTGCCTAACGTCCACTGGCCAGCCTGATTTCCATTGA

>LOC_Os08g34050.2; OsPFK07_mod_FR13A

ATGGCTGTTTCTTTAAAATCAAGTGGCAGTTTTTGTAGCACACCGCCTCAGTGGCTGCATTCAACAAGGGATCGAATTTTATACGGTTATTCTCATTCAAATGCCAAAGAGTGCACTTGCAAGAAAACAAAAAGGCCTGCTCCACTGTGTGTTAAAGCTACTTCCACGAAAGTGGAATTAGATTTCAATGATCCATCTTGGAAGCAGAAGTTTCAGGAAGACTGGGATAAACGTTTTAATTTGCCACGTATTACAGACATATATGATTTGAAACCAAGGCCAACCACATTCTCACTCAAGAAAAACAGAAGTCCTGCAGGTGATGAAAATGGTACACCTATGGATAAATGGAATGGTTATGTGAACAGCGATGATCGAGCACTTTTGAAGGTGATAAAGTATTCCTCGCCTAACTCTGCTGGAGCAGAGTGCATTGATCCTGACTGTAGCTGGGTGGAACAATGGGTACATCGTGCAGGCCCTCGTAAGGAGATATACTATGAACCAGAGGAAGTAAAAGCTGCCATAGTTACTTGTGGAGGGCTCTGCCCTGGTTTGAACGATGTCATCAGACAGATAGTATTCACTCTAGAGACCTATGGGGTTAAGAATATTGTTGGGATTCCATTTGGTTATCGTGGATTTTTTGAAAAGGGCCTAAAAGAAATGCCACTTTCACGTCATCTGGTGGAGAACATAAATCTTGCTGGTGGAAGTTTTCTAGGAGTCTCTCGTGGAGGAGCTAAAACTAGTGAGATTGTAGATAGTATACAGGCCAGAAGAATTGATATGCTTTTCGTGCTTGGAGGAAATGGTACCCATGCAGGAGCAAATGCTATCCATGAAGAGTGCCGGAAGAGAAAGCTAAAAGTTTCAGTTGTAGCAGTTCCAAAGACCATCGACAATGATATACTTTTGATGGACAAAACATTTGGTTTCGATACGGCTGTTGAAGAAGCTCAGCGGGCCATTAATTCTGCATATATAGAGGCACGAAGCGCATACCATGGCATTGGTTTGGTCAAATTAATGGGAAGAAGCAGTGGCTTCATTGCAATGCATGCTTCCCTTTCAAGTGGGCAGGTTGATGTCTGTTTAATACCAGAGGTTCCTTTCACGCTTGATGGAGAATATGGTGTTCTACGACACCTTGAGCATTTGTTAAAGACCAAAGGATTCTGTGTTGTTTGTGTTGCTGAAGCTGCAGGACAAGAGTTATTACAAAAATCAGGTGCAACAGATGCATCTGGAAATGTGATACTTAGTGACATCGGTGTTCATATGCAACAGAAGATTAAGATGCATTTCAAGGACATTGGTGTTCCTGCTGATGTAAAATACATTGATCCGACATATATGGTTCGGGCATGTCGTGCCAATGCATCTGATGCAATTTTGTGCACTGTACTTGGACAAAATGCTGTCCATGGAGCATTTGCTGGGTTCAGTGGCATCACTTCTTGCATCTGCAACACGCACTACGTCTACCTCCCCATCACAGAAGTCATAACAGTACCGAAGCGCGTGAACCCTAATAGCAGGATGTGGCACCGTTGCCTAACGTCCACTGGCCAGCCTGATTTCCATTGA

>LOC_Os08g34050.2; OsPFK07_mod_Nipponbare

MAVSLKSSGSFCSTPPQWLHSTRDRILYGYSHSNAKECTCKKTKRPAPLCVKATSTKVELDFNDPSWKQKFQEDWDKRFNLPRITDIYDLKPRPTTFSLKKNRSPAGDENGTPMDKWNGYVNSDDRALLKVIKYSSPNSAGAECIDPDCSWVEQWVHRAGPRKEIYYEPEEVKAAIVTCGGLCPGLNDVIRQIVFTLETYGVKNIVGIPFGYRGFFEKGLKEMPLSRHLVENINLAGGSFLGVSRGGAKTSEIVDSIQARRIDMLFVLGGNGTHAGANAIHEECRKRKLKVSVVAVPKTIDNDILLMDKTFGFDTAVEEAQRAINSAYIEARSAYHGIGLVKLMGRSSGFIAMHASLSSGQVDVCLIPEVPFTLDGEYGVLRHLEHLLKTKGFCVVCVAEAAGQEFTKIRCNRCIWKCDT**HRCSYATED*DAFQGHWCSC*CKIH*SDIYGSGMSCQCI*CNFVHCTWTKCCPWSICWVQWHHFLHLQHALRLPPHHRSHNSTEAREP**QDVAPLPNVHWPA*FPLX

>LOC_Os08g34050.2; OsPFK07_mod_FR13A

MAVSLKSSGSFCSTPPQWLHSTRDRILYGYSHSNAKECTCKKTKRPAPLCVKATSTKVELDFNDPSWKQKFQEDWDKRFNLPRITDIYDLKPRPTTFSLKKNRSPAGDENGTPMDKWNGYVNSDDRALLKVIKYSSPNSAGAECIDPDCSWVEQWVHRAGPRKEIYYEPEEVKAAIVTCGGLCPGLNDVIRQIVFTLETYGVKNIVGIPFGYRGFFEKGLKEMPLSRHLVENINLAGGSFLGVSRGGAKTSEIVDSIQARRIDMLFVLGGNGTHAGANAIHEECRKRKLKVSVVAVPKTIDNDILLMDKTFGFDTAVEEAQRAINSAYIEARSAYHGIGLVKLMGRSSGFIAMHASLSSGQVDVCLIPEVPFTLDGEYGVLRHLEHLLKTKGFCVVCVAEAAGQELLQKSGATDASGNVILSDIGVHMQQKIKMHFKDIGVPADVKYIDPTYMVRACRANASDAILCTVLGQNAVHGAFAGFSGITSCICNTHYVYLPITEVITVPKRVNPNSRMWHRCLTSTGQPDFH*

>LOC_Os09g24910.2; OsPFK08_mod

ATGACCTTTTCTGGGATGGACATTGCTTTAAAAGCAAGCACACACTCTTCTACATCCCAGCAACACTGGTTGCATTCAACCAGGTACCGGTGTCAATATGGTTTGGGTTCCACTCACTTGAATGGAAGAAAGAGAAGTCCTATGGTACTGTCTGTAAGAGCTGTTTCTGGGAAATCAGACTTAGATTTCAGTGATCCTTCTTGGAAGGAAAAGTATCAAGAAGACTGGAATAGGCGTTTCAGTTTGCCGCATATTACAGATATATATGATTTGAAGCCAAGGCTAACTACATTCTCTCTGAAGAAAAACAGGACTGATGGTGGTAGTTTATCAGCAGATAAGTGGAATGGCTATGTAAATAAGGATGACCGTGCACTTCTGAAGGTGATAAAGTATGCCTCCCCTACTTCTGCTGGAGCTGAGTGCGTAGATCCTGACTGCAGTTGGGTTGAACATTGGATTCATCGTGCAGGGCCTCGTAAGGAGATATACTATGAGCCTGCAGAAGTAAAAGCTGCTATTGTTACCTGTGGAGGCCTCTGCCCTGGTTTAAATGATGTCATTAGACAGATAGTATTTACATTGGAGATCTATGGGGTTAAGAACATTGTTGGAATTCAGTTTGGTTATCGTGGATTTTTTGAGAAAGGCTTAAAAGAAATGCCTCTTTCACGTAAAGTGGTGGAAAACATAAATCTTTCTGGTGGAAGTTTCCTAGGTGTGTCTCGTGGAGGAGCTAAAACTAGTGAGATCGTCGATAGTATACAAGCCAGAAGAATTGATATGCTTTTTGTAATTGGTGGAAACGGTAGCCATGCAGGAGCTAATGCTATCCATGAGGAGTGTCGTAAGAGAAAACTGAAAGTGTCAGTTGTAGCAGTTCCAAAGACAATTGATAATGATATACTATTCATGGATAAGACTTTTGGTTTTGACACGGCTGTAGAAGAAGCTCAGCGTGCCATCAATTCTGCCTACATAGAGGCACGAAGTGCATATCATGGAATTGGGTTGGTCAAATTAATGGGAAGAAGTAGTGGGTTCATTGCCATGCAAGCTTCTCTTTCCAGTGGACAGATTGATGTCTGCCTAATACCCGAGGTATCTTTTACACTAGATGGAGAACATGGTGTCATGCGACACCTTGAACATTTACTGGAAAAAAAGGGATTTTGCGTGGTTTGTGTTGCTGAAGGTGCAGGGCAGGATTTACTGCAAAAATCAAATGCAACTGATGCATCAGGAAATGTAATACTTAGTGACTTTGGTGTCCACATGCAACAGAAGATTAAGAGTCATTTCAAGGACATCGGTGTTCCAGCTGATGTAAAATACATTGATCCGACATATATGGTCCGGGCCTGTCGTGCGAATGCATCTGATGCTATCTTGTGCACTGTACTTGGACAAAATGCTGTTCATGGAGCCTTTGCCGGGTTCAGTGGTATCACATCTGGTATTTGCAACACGCACTACGCTTTCCTCCCGATCACAGAAGTCATCACAAAACCAAAGCGCGTGAACCCCAACAGCAGGATGTGGCACCGCTGCCTCACTTCCACTGGCCAACCGGACTTCCACTGA

>LOC_Os09g24910.2; OsPFK08_mod

MTFSGMDIALKASTHSSTSQQHWLHSTRYRCQYGLGSTHLNGRKRSPMVLSVRAVSGKSDLDFSDPSWKEKYQEDWNRRFSLPHITDIYDLKPRLTTFSLKKNRTDGGSLSADKWNGYVNKDDRALLKVIKYASPTSAGAECVDPDCSWVEHWIHRAGPRKEIYYEPAEVKAAIVTCGGLCPGLNDVIRQIVFTLEIYGVKNIVGIQFGYRGFFEKGLKEMPLSRKVVENINLSGGSFLGVSRGGAKTSEIVDSIQARRIDMLFVIGGNGSHAGANAIHEECRKRKLKVSVVAVPKTIDNDILFMDKTFGFDTAVEEAQRAINSAYIEARSAYHGIGLVKLMGRSSGFIAMQASLSSGQIDVCLIPEVSFTLDGEHGVMRHLEHLLEKKGFCVVCVAEGAGQDLLQKSNATDASGNVILSDFGVHMQQKIKSHFKDIGVPADVKYIDPTYMVRACRANASDAILCTVLGQNAVHGAFAGFSGITSGICNTHYAFLPITEVITKPKRVNPNSRMWHRCLTSTGQPDFH*

>LOC_Os09g12650.2; PFPA4_mod

ATGTCCATGAACGCGGACCTCGGCAAGCCGAGGGAGCTCACCGGGTTGCAGCAGAGGAGGGCTCTGTATCAGCCGGAGCTGCCTCCATGCCTTGAGTTTTTCAACCAACATGTTCAGGGAAAGGCCATCAGAGTGGAGTTTGGCGATTCGACGACGACCATCGACCCGACATGCGCAAATATGGTTGTGCAGGAATTTCCCAACACCTTTGGTCAGCCTCTGGTACATTTCCTGAAGCCGAACAAGATGGATGCGCAAGCCAACGATGAACATCCACCAATCAGGGTTGGTGTGGTGTTCTCTGGGAGGCAATCGCCAGGGGGGCACAATGTGATATGGGGCATCTATGATGCTATGAAAACTCAAAATCTACAAAGTGTTTTGCTTGGATTTATTGGTGGCACAGAAGGCCTATTTGCAAATCAGACATTGGAGATCACAGATGATGTTCTTTCAGCATATAGAAACCAAGGTGGTTTTGATTTTCTCGGCAGAACTGTTGATCAAATCCACACAACTGAGCAAGTGAATGCTGCAATGTCAACATGCTGTGATCTTGACTTGGATGGCCTCGTCATCATTGGAGGGGTGACCTCCAATTCAGATGCTGCTCAGCTTGCAGAAACATTTGCCAATCACAACTGCAAGACAAAGGTTGTAGGCGTGCCTGTTTCATTAAATGGTGATCTCAAGAATCAGTTTGTTGAGACAACTGTTGGGTTTGATACAGTGTGCAAGGTGAATTCCCAGCTTATAAGTAATGTTTGCCTCGATGCAATCTCAGCTGGGAAGTACTACCATTTTGTCCGTGTGATGGGTTGGAAAGCATCTCATGTTGCCTTGGAGTGTGCACTTCAATCACAACCAAATATGGTTATTTTAGGGGAAGAAGTGGCATTTTCCAAGCTCACTTTGAAGGAAATTATAAGCAAGATATGTGATGGAGTGCAAGCAAGGGCAGCACAAGAGAAGTACCATGGTGTATTACTTATTTCGGAGGGACTAATCGAAAGCATTCCAGAAATGTTTGCACTTATTCAGGAAATTAATATTCTCCACAGCAATAAAGTTCCTGAGAACAACATTCCATCTCAACTTTCTCCATGGGCTACTGCCTTGTATAATTACTTGCCTCCTTTCATAAGAAGAGAGTTGCTGCTACATCAAGACTCCGATAACTCAGCACAATTGTCCCAGATTGATACTGAGCAACTCTTAGCTCATTTAGTAGAAGCAGAAATGAACAAGCGAATGAAAGAGGGGAAATACATTGGAAGGAAATTCAGCTCCGTTTGCCACTTTTTCGGGTATCAAGCGCGAGGATCCTTACCATCAAACTTCGATTGTGATTACGCCTATGTTCTTGGTCATATCTGCATGCACATACTAGCAGCTGGGCTGAATGGTTACATGGCTTTTGCAACAAATCTGAAGGAACCAACAAACAAATGGCGATGCGCTGCCGTTCCTCTAACAGCAATGATGAGTGTGAAGAGGCACTCACGCAGTCCTGGAGCTGTTCCTACTGGAAAGCCAGTTATCCATCCTTCTCCAGTTGACCTGCAAGGGAAGGCTTATGCGCTCCTGAGAGAGAAAGCCTCGAGCTTCCTATTAGATGATTTCTACAGAACTCCAGGGGGCATTCAATTTGATGGATCTGGAACAAATGTTAAGCCAATCACACTGACTGTCGAAGACCAAGACTACCTGGGTGACATTGAGCTACTGCAAGACTACTTGGAGAAGGTGAGGAACATTGTGAAGCCCGGATGCTCGAGGGAGATCCTGAAGGCGGCGATAAGCTCGATGTCATCAGTGAAGGATGTGCTGAAGGTTATGTCGGCTCCCTTCTATGCAGAGCTCCCTCTGTTCAATCTGAACTGA

>LOC_Os09g12650.2; PFPA4_mod MSMNADLGKPRELTGLQQRRALYQPELPPCLEFFNQHVQGKAIRVEFGDSTTTIDPTCANMVVQEFPNTFGQPLVHFLKPNKMDAQANDEHPPIRVGVVFSGRQSPGGHNVIWGIYDAMKTQNLQSVLLGFIGGTEGLFANQTLEITDDVLSAYRNQGGFDFLGRTVDQIHTTEQVNAAMSTCCDLDLDGLVIIGGVTSNSDAAQLAETFANHNCKTKVVGVPVSLNGDLKNQFVETTVGFDTVCKVNSQLISNVCLDAISAGKYYHFVRVMGWKASHVALECALQSQPNMVILGEEVAFSKLTLKEIISKICDGVQARAAQEKYHGVLLISEGLIESIPEMFALIQEINILHSNKVPENNIPSQLSPWATALYNYLPPFIRRELLLHQDSDNSAQLSQIDTEQLLAHLVEAEMNKRMKEGKYIGRKFSSVCHFFGYQARGSLPSNFDCDYAYVLGHICMHILAAGLNGYMAFATNLKEPTNKWRCAAVPLTAMMSVKRHSRSPGAVPTGKPVIHPSPVDLQGKAYALLREKASSFLLDDFYRTPGGIQFDGSGTNVKPITLTVEDQDYLGDIELLQDYLEKVRNIVKPGCSREILKAAISSMSSVKDVLKVMSAPFYAELPLFNLN*

**Supplemental Figure 3:** Subcellular localization of PFK isoforms from rice. Full-length mRNA sequences or the N-terminal part of the sequences were cloned in frame in front of a GFP-coding sequence and were transiently transformed into tobacco leaves by Agrobacterium infiltration. After 3-4 days, protoplasts or undigested leaf discs were isolated and analyzed by confocal microscopy. Green color represents GFP fluorescence, red color represents chlorophyll autofluorescence. Full-length CDS: A, OsPFK01; B, OsPFK02; C, OsPFK04; D, OsPFK05; E, OsPFK06; I, J, OsPFK03; O, OsPFK09; S, OsPFK08; T, OsPFK10. N-terminal part: F-H, OsPFK03; K-N, OsPFK09; P-R, OsPFK05.

**Supplemental Figure 4:** Expression of the PFK_B group members in different organs. eFP browser pictures were obtained through http://bar.utoronto.ca (Winter et al., 2007). Red color intensity shows high expression level in the respective tissue type.
